# Lactylation of HSC70 Impairs Chaperone‐Mediated Autophagy to Promote Pyrin Inflammasome‐Dependent PANoptosis in Macrophages During Sepsis

**DOI:** 10.1002/advs.78586

**Published:** 2026-11-03

**Authors:** Ying Chen, Yan Zhang, Pei Wang, Jiamin Ma, Xinru Zhao, Yi Li, Zhenghao Deng, Xueqing Meng, Jiali Zhu, Liangfang Yao, Jinbao Li

**Affiliations:** ^1^ Department of Anesthesiology Shanghai General Hospital Shanghai Jiao Tong University School of Medicine Shanghai China; ^2^ Department of Anesthesiology and Perioperative Medicine General Hospital of Ningxia Medical University Yinchuan China; ^3^ School of Anesthesiology Shandong Second Medical University Shandong China; ^4^ Department of Anesthesiology The Affiliated Hospital Southwest Medical University Luzhou Sichuan China

**Keywords:** CMA, lysine lactylation, macrophage, PANoptosis, sepsis

## Abstract

Sepsis‐related cytokine storm is associated with poor prognosis in septic patients. IFNγ/LPS can synergistically trigger a sepsis‐associated proinflammatory cytokine storm and induce macrophage death in the form of PANoptosis. The ensuing release of proinflammatory cytokines further amplifies macrophage death, leading to subsequent multiple organ damage. Concurrently, the IFNγ/LPS induced inflammatory cascade also downregulates chaperone‐mediated autophagy (CMA) activity, and this dysregulation further exacerbates the inflammatory response in macrophages and in vivo. Thus, a positive feedback loop between dysfunctional CMA and the inflammatory cascade is established. Enhancing CMA activation reduces macrophage death and organ damage, whereas inhibiting CMA exacerbates macrophage PANoptosis and organ injury. Mechanistically, impaired CMA leads to accumulation of the transcription factor CUX1, which subsequently upregulates Pyrin expression and promotes Pyrin‐mediated PANoptosome assembly, thereby driving macrophage PANoptosis. In addition to the cytokine storm, the lacylation at lysine 561 (K561) of HSC70, a core component of the CMA machinery, is associated with reduced CMA activity. Blocking lactylation can similarly attenuate macrophage death and multiple organ injury in sepsis. Taken together, this “Kla‐CMA‐CUX1/Pyrin‐PANoptosis” signaling axis, linking metabolic reprogramming to immune dysregulation in sepsis, suggests that targeting key nodes of this axis may represent a promising therapeutic strategy to reduce sepsis‐associated mortality.

## Graphical abstract

In sepsis, lactate accumulation drives HSC70 K561 lactylation, which impairs chaperone‐mediated autophagy and blocks CUX1 degradation. Accumulated CUX1 transcriptionally upregulates Pyrin, triggering PANoptosis in macrophages and exacerbating inflammatory storm and organ injury. Targeting lactate production or activating CMA relieves this pathological cascade, providing potential therapeutic strategies for sepsis.

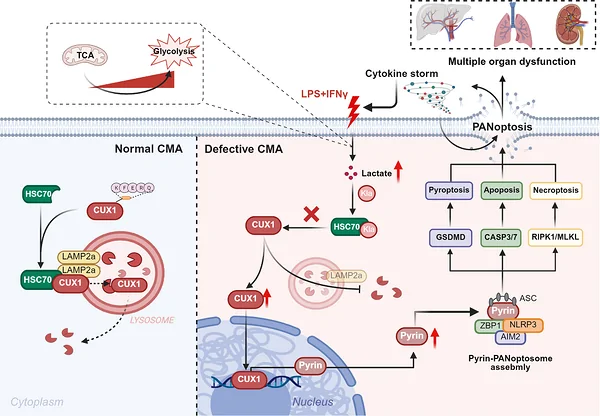

## 1 Introduction

Sepsis is a life‐threatening syndrome of multiple organ dysfunction resulting from a dysregulated host immune response to infection [1]. It is primarily characterized by uncontrolled hyperinflammation driven by the excessive release of proinflammatory cytokines, a phenomenon commonly referred to as a cytokine storm. This overwhelming cytokine response, which produces capillary leakage and microcirculatory collapse, can involve the lung, kidney, and liver, and may even lead to multiple organ dysfunction and failure [1, 2, 3], serving as the primary cause of death in septic patients. Although appropriate antibiotic therapy can alleviate certain aspects of sepsis, the overall mortality rate remains above 20% [1, 4, 5, 6, 7, 8], placing a substantial burden on families and healthcare systems worldwide. Despite advances in supportive care, the mechanisms linking the initial inflammatory surge to end‐organ injury remain incompletely understood, hindering the development of targeted therapeutic interventions.

Macrophages are central effectors of the innate immune system, playing critical roles in cytokine secretion, antigen presentation, and phagocytosis [9, 10, 11]. As such, they are essential for the establishment and preservation of immune homeostasis. The cytokine storm accompanied by coronavirus disease 2019 (COVID‐19) infection can predispose macrophages to inflammatory cell death, thereby contributing to multiple organ dysfunction [12]. In sepsis, a key driver of organ damage is inflammatory programmed cell death triggered by the cytokine storm [13, 14]. Beyond classical apoptosis, necroptosis, and pyroptosis, a novel integrated mode of inflammatory cell death known as PANoptosis has recently been identified [12]. PANoptosis is orchestrated by a multi‐protein complex called the PANoptosome, a critical event for PANoptosis initiation. This complex is mediated by sensor proteins, including absent in melanoma 2 (AIM2), nucleotide‐binding oligomerization domain (NOD)‐like receptor pyrin domain‐containing 3 (NLRP3), Pyrin (coded by the gene Mediterranean fever (*MEFV*)), and Z‐DNA binding protein 1 (ZBP1), which integrates components from distinct cell death pathways and is activated by specific triggers such as pathogen‐associated molecular patterns (PAMPs) and cytokines [12, 15, 16, 17, 18, 19]. PANoptosis culminates in lytic cell death and the release of proinflammatory intracellular contents, thereby amplifying local inflammation and exacerbating tissue injury, particularly in vital organs. Therefore, the elucidation of key signaling pathways that trigger macrophage PANoptosis, along with the identification of potential therapeutic targets, is crucial for inhibiting dysregulated PANoptosis, ameliorating sepsis‐associated organ injury, and ultimately improving patient prognosis.

Chaperone‐mediated autophagy (CMA) is a selective lysosomal degradation process responsible for maintaining cellular proteostasis by targeting specific proteins for degradation [20]. A central component of the CMA pathway, heat shock cognate protein 70 (HSC70/HSPA8), is indispensable for this process, as it recognizes cargo proteins bearing a Lys‐Phe‐Glu‐Arg‐Gln (KFERQ)‐like motif and shuttles them to the lysosomal membrane for translocation [20, 21]. Subsequently, mediated by lysosome‐associated membrane protein 2a (LAMP2a) [22], substrate proteins enter lysosomes for unfolding and degradation. The regulatory role of CMA in inflammatory processes is increasingly recognized. Previous studies have established that CMA activity plays a critical role in modulating the activation of the canonical inflammasome, such as NLRP3, in various diseases [23, 24]. While inflammatory signals can impair CMA activity [25, 26], it remains unknown whether and how sepsis‐related cytokine storm reprograms CMA. Moreover, the specific contribution of CMA to PANoptosome assembly and macrophage PANoptosis during sepsis has yet to be explored. Elucidating this mechanism may uncover a novel immunometabolic axis underlying septic immunopathology and offer potential therapeutic strategies for immune dysregulation.

The dysregulated immune response characteristics of the sepsis‐related cytokine storm are inextricably linked to profound metabolic reprogramming within immune cells. This hyperinflammatory state is fueled by a shift toward glycolysis, resulting in the massive accumulation of lactate [27]. Serving as a precursor for lysine lactylation (Kla), lactate facilitates widespread protein modifications that extend beyond the histone to encompass diverse functional proteins, thereby influencing a spectrum of cellular processes. Given that lactate levels are robustly elevated in sepsis, increased protein lactylation has been correlated with disease severity [28, 29, 30]. While emerging evidence has begun to shed light on the role of lactylation in modulating macrophage polarization [31] and neuroinflammation [32, 33], whether and how lactylation regulates CMA activity in macrophages and the mechanism by which inflammatory signaling and cellular fate are linked by CMA have not been fully elucidated. Thus, elucidating whether sepsis‐induced lactylation impairs CMA function via modification of key degradation pathway components represents a critical mechanistic question, the answer to which may uncover novel aspects of immune dysregulation during the sepsis‐associated cytokine storm.

In this study, we aim to elucidate how CMA regulates PANoptosome formation for the alteration of macrophage fate in the context of sepsis. We demonstrated that the sepsis‐associated cytokine storm could significantly suppress CMA activity in macrophages both in vitro and in vivo. This impairment drove excessive macrophage PANoptosis, thereby contributing to subsequent multi‐organ injury, and this pathological cascade could be largely attenuated by pharmacological CMA activation. Mechanistically, we linked CMA suppression to sepsis‐induced metabolic rewiring, identifying lysine lactylation of the core CMA chaperone HSC70 as the causal post‐translational modification underlying CMA dysfunction. Generally, HSC70 lactylation impaired CMA activity, leading to increased stabilization of the transcription factor CUX1 (Homeobox protein cut‐like 1), subsequent activation of the Pyrin inflammasome, and ultimately macrophage PANoptosis. Collectively, our findings define a pathogenic “lactylation‐CMA‐PANoptosis” axis in sepsis, illustrating how sepsis‐associated metabolic changes are translated into sustained inflammatory signaling and tissue damage. Unraveling this cascade provides crucial insight into the pathogenesis of sepsis‐induced organ failure and highlights potential therapeutic strategies, such as inhibiting HSC70 lactylation or enhancing CMA flux, to mitigate organ injury in this critical condition.

## 2 Results

### 2.1 IFNγ Induces LPS‐Activated Macrophage Death by Promoting Sepsis‐Associated Inflammatory Storm, Exacerbating Multi‐Organ Injury

The cytokine storm is a life‐threatening pathological condition characterized by uncontrolled immune hyperactivation, excessive release of proinflammatory cytokines, systemic inflammation, and a high risk of multi‐organ failure. To investigate the relationship between the inflammatory storm and poor prognosis in sepsis, we initially measured the plasma levels of proinflammatory cytokines in cecal ligation and puncture (CLP)‐induced septic mice. The experimental design, including the modeling and sampling scheme, is illustrated in Figure S1A. The results showed that the levels of various inflammatory cytokines (IL‐1β, TNF‐α, IFNγ, IL‐12, IL‐6, and IL‐10) were markedly elevated in the CLP‐induced septic mouse model (Figure 1A), indicating CLP could trigger a cytokine storm in mice. We also detected the percentage of SYTOX‐positive (indicative of cell death) peripheral blood mononuclear cells (PBMCs) by flow cytometry and confirmed the increased deaths of macrophages in murine CLP models (Figure 1B,C). Furthermore, histopathological analysis of multiple organs via hematoxylin and eosin (H&E) staining showed prominent pathological changes in CLP mice, including increased inflammatory cell infiltration and alveolar septal thickening in the lung, disorganization of hepatic cords in the liver, and loss of the brush border with tubular dilation in the kidney (Figure 1D). Consistent with these morphological alterations, pulmonary assessment via wet/dry weight ratio analysis, as well as key biochemical indicators of hepatic and renal dysfunction (ALT, AST, Cr, and BUN), were significantly elevated in septic mice (Figure S1B–D), accompanied by a significantly reduced survival rate (Figure S1E). Hence, we establish a plausible correlation between the cytokine storm, striking macrophage deaths, and extensive multi‐organ damage in CLP‐induced septic mice.

**FIGURE 1:**
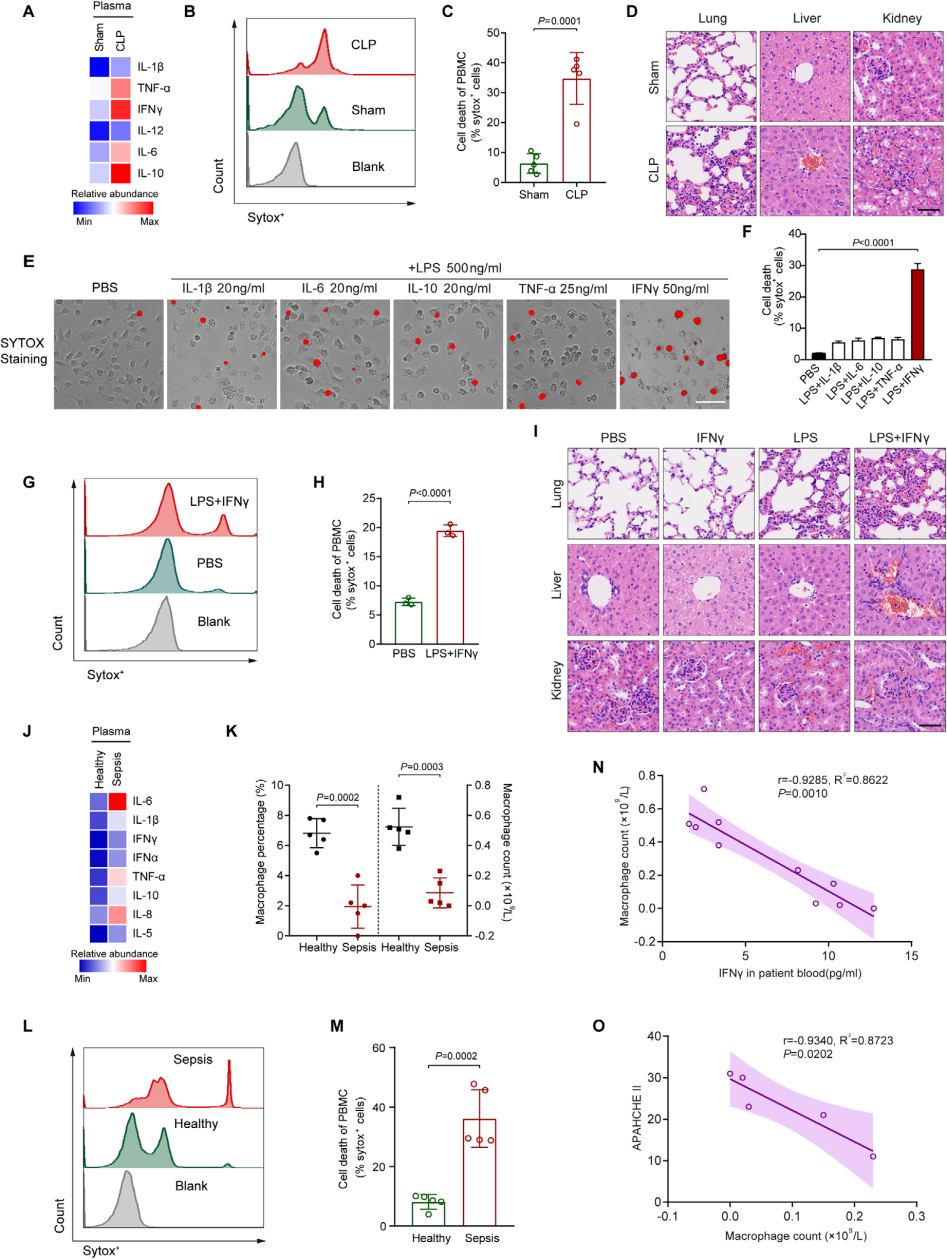
Sepsis‐related inflammatory cytokine storm induces macrophages death and multiple organ damage. (A) Heatmap depicting the levels of proinflammatory cytokines in plasma of sham and CLP treated mice 6 h after modelling. (B,C) Percent of peripheral blood mononuclear cells (PBMCs) that are dead 6 h after CLP in mice using SYTOX staining detected by flow cytometer compared with sham controls (n = 5). (D) Representative HE staining images from lung, liver and kidney tissues isolated from sham and CLP mice. Scale bar = 50 µm. (E,F) Percent of bone marrow derived macrophages (BMDMs) death 24 h after treatment with LPS coupled with various proinflammatory cytokines (IL‐1β, IL‐6, IL‐10, TNF‐α and IFNγ) using SYTOX staining (n = 3). Scale bar = 50 µm. (G,H) Percent of PBMCs that are dead 24 h after LPS+IFNγ intraperitoneal injection in mice using SYTOX staining detected by flow cytometer compared with PBS‐injected controls (n = 3). (I) Representative HE staining images of lung, liver and kidney tissues isolated from PBS, IFNγ, LPS or LPS+IFNγ injected mice. Scale bar = 50 µm. (J) Heatmap depicting the level of multiple inflammatory cytokines in the plasma of healthy and sepsis patients. (K) Comparison of percent and count of macrophages between healthy and sepsis patients (n = 5). (L–M) Percent of PBMCs that are dead detected in sepsis patients using SYTOX staining detected by flow cytometer compared with healthy patients (n = 5). (N) Correlation analysis between the level of IFNγ and the macrophages count in the blood of healthy and sepsis patients (n = 10). (O) Correlation analysis between the macrophages counts and APACHE II score from sepsis patients (n = 5). Data are shown as mean ± SD. Analysis was performed using the two‐tailed Student's *t*‐tests (C, H, K and M), one‐way ANOVA (F) or Pearson's correlation (N and O).

To establish a causal link between the inflammatory storm and macrophage death, we first explored this relationship in vitro. Screening of various proinflammatory cytokines revealed that IFNγ, in combination with LPS, induced the most significant cell death in bone marrow‐derived macrophages (BMDMs) (Figure 1E,F). Subsequently, we sought to validate the proinflammatory and cytotoxic effects of this combination in vivo. Intraperitoneal injection of LPS and IFNγ into mice resulted in elevated serum levels of IL‐1β, IL‐6, and TNF‐α (Figure S1F), an increased proportion of SYTOX‐positive PBMCs (Figure 1G,H), and the most severe organ damage as assessed by H&E staining (Figure 1I), pulmonary wet/dry ratio, and biochemical indicators (Figure S1G–I). Consistent with the exacerbated pathology, mice receiving LPS and IFNγ exhibited the lowest survival probability compared to PBS, IFNγ, or LPS‐treated groups (Figure S1J). So far, we reaffirm the correlated proinflammatory cytokine storm, macrophage deaths, and multi‐organ dysfunction in LPS‐ and IFNγ‐injected mice.

Furthermore, we sought to validate this correlation in clinical patients. Peripheral blood specimens from septic patients and healthy donors were analyzed. Compared with healthy controls, septic patients displayed markedly elevated levels of multiple proinflammatory cytokines, accompanied by a significant reduction in both the percentage and absolute count of peripheral blood macrophages (Figure 1J,K). Correlation analysis further revealed a negative correlation between peripheral macrophage counts and serum IFNγ levels in these patients (Figure 1N). In addition, flow cytometric analysis also showed a higher percentage of SYTOX‐positive PBMCs in septic patients, indicating increased cell death (Figure 1L,M). Moreover, macrophage counts were negatively correlated with the APACHE II score (Figure 1O). These clinical observations suggest that IFNγ may contribute to sepsis pathophysiology by associating with macrophage death. Collectively, IFNγ serves as a critical driver of sepsis pathogenesis by synergizing with LPS to trigger a fulminant cytokine storm, which directly induces macrophage death, thereby promoting immune dysfunction and contributing to lethal multi‐organ failure.

### 2.2 IFNγ/LPS Co‐Stimulation Triggers PANoptosis in Macrophages

Macrophages play a dual role in the progression of sepsis [34]. Although essential for pathogen clearance, their excessive activation can trigger uncontrolled cytokine release, leading to tissue damage [35, 36]. To investigate how the cytokine storm influences macrophage fate, we mimicked the septic inflammatory storm by exposing BMDMs to a combination of LPS and IFNγ. The results showed that this co‐stimulation led to massive death of BMDMs in a concentration‐ and time‐dependent manner (Figure 2A,B and Figure S2A,B). Consistent with SYTOX Green‐based quantification of cell death, lactate dehydrogenase (LDH) release‐an indicator of cytotoxicity‐also increased in a concentration‐ and time‐dependent manner (Figure S2D,E), while CCK‐8 assays revealed a concomitant reduction in BMDM viability (Figure S2C). Furthermore, IFNγ/LPS co‐stimulation successfully recapitulated a proinflammatory cytokine storm in vitro, characterized by a significant upregulation of IL‐1β, IL‐6, and TNF‐α (Figure 2C and Figure S2F). Collectively, these findings confirm that the combined treatment effectively induces macrophage death and inflammatory activation.

**FIGURE 2:**
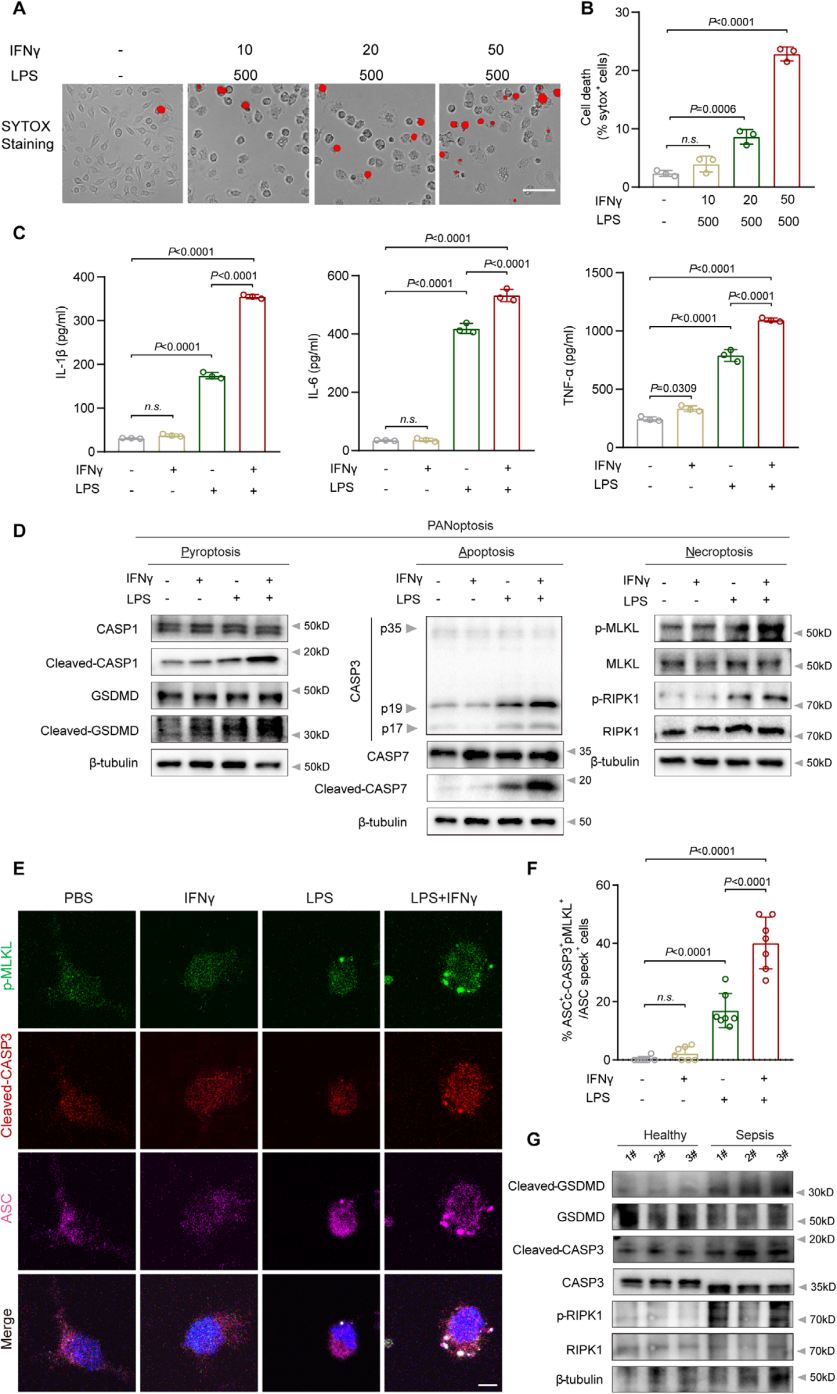
LPS and IFNγ induces septic inflammatory cytokine storm and PANoptosis in macrophages. (A,B) Percent of BMDMs that are dead under the stimulation of LPS+IFNγ with indicated concentration for 24 h using SYTOX staining (n = 3). Scale bar = 50 µm. (C) Elisa assay of IL‐1β, IL‐6 and TNF‐α in the supernatants of BMDMs treated with PBS, IFNγ, LPS or LPS+IFNγ (n = 3). (D) Immunoblotting analysis of pro‐ and cleaved caspase‐1 (CASP1), pro‐ and cleaved GSDMD; pro‐ (P35) and cleaved (P17/19) caspase‐3 (CASP3), pro‐ and cleaved caspase‐7 (CASP7); phosphorylated RIPK1 (pRIPK1) and total RIPK1 (tRIPK1), phosphorylated MLKL (pMLKL) and total MLKL (tMLKL) in PBS, IFNγ, LPS or LPS+IFNγ treated BMDMs. (E) Representative immunofluorescence images of ASC, c‐CASP3, and pMLKL in BMDMs treated with PBS, IFNγ, LPS or LPS+IFNγ for 24 h. White dots indicate the ASC speck. Scale bar = 5 µm. (F) Quantification of the percentage of cells with ASC^+^c‐CASP3^+^pMLKL^+^ specks among the ASC speck^+^ cells (n = 7). (G) Immunoblotting analysis of pro‐ and cleaved GSDMD, pro‐ and cleaved CASP3, pRIPK1 and tRIPK1 in PBMCs in sepsis and healthy patients. Data are representative of at least three independent experiments. Data are shown as mean ± SD. Analysis was performed using one‐way ANOVA (B‐C and F). *n.s*. stands for not significant.

To delineate the specific mode of cell death in BMDMs, we investigated the occurrence of PANoptosis, a unified inflammatory programmed cell death pathway that integrates markers of pyroptosis, apoptosis, and necroptosis. Immunoblot analysis confirmed that IFNγ/LPS co‐stimulation induced cleavage of both caspase‐1 (CASP1) and Gasdermin D (GSDMD) (pyroptosis), activation of caspase‐3 (CASP3)/caspase‐7 (CASP7) (apoptosis), and phosphorylation of receptor interacting serine/threonine protein kinase 1 (RIPK1) and mixed lineage kinase domain‐like pseudokinase (MLKL) (necroptosis) (Figure 2D). Furthermore, immunofluorescence staining visualized the colocalization of apoptosis‐associated speck‐like protein containing a CARD (PYCARD/ASC) speck with cleavage of CASP3 (c‐CASP3, indicative of apoptosis, red), and phosphorylated MLKL (p‐MLKL, indicative of necroptosis, green) (Figure 2E), leading to an increase in the percentage of ASC^+^c‐CASP3^+^pMLKL^+^ specks (Figure 2F), indicating an enhanced inflammasome assembly and PANoptosis execution. These results demonstrate that LPS and IFNγ synergistically trigger PANoptosis in BMDMs, leading to inflammatory cell death. We next investigated whether the increased PBMCs death observed in septic patients with inflammatory storm was also dependent on PANoptosis. Immunoblot analysis revealed that key PANoptosis executioners were significantly elevated in septic patients compared with controls (Figure 1G), confirming that PANoptosis is exacerbated in patients with sepsis‐related proinflammatory cytokine storm.

### 2.3 CMA Downregulation Upon IFNγ/LPS Co‐Stimulation Induces PANoptosis in Macrophages

To elucidate the mechanism underlying the robust cell death during the sepsis‐related inflammatory storm, we performed RNA sequencing on BMDMs treated with PBS, LPS, or LPS plus IFNγ. Kyoto Encyclopedia of Genes and Genomes (KEGG) enrichment analysis of differentially expressed genes (DEGs) indicated that co‐stimulation with LPS and IFNγ significantly affected several pathways, including Autophagy, Phagosome, and Lysosome function (Figure 3A,B). However, heatmap analysis of RNA‐seq data showed no remarkable changes in the mRNA levels of most autophagy‐associated genes across the three groups (Figure 3C). All lysosome‐, autophagy‐, and phagosome‐related genes detected in our RNA‐seq data were presented in Table S1. Consequently, we examined key autophagy markers using immunoblotting and quantitative real‐time polymerase chain reaction (qPCR). Screening results showed that IFNγ/LPS co‐stimulation specifically reduced the abundance of two core components of chaperone‐mediated autophagy (CMA), LAMP2a and HSC70, without affecting other autophagy‐related proteins (Figure 3D). However, their mRNA levels remained unchanged, suggesting that this regulation might occur at the post‐transcriptional level (Figure S3A–C). This supports the specificity of the CMA‐dependent mechanism. Given the central role of LAMP2a and HSC70 in CMA, we focused subsequent analyses on this selective autophagy pathway. To assess CMA activity directly, we performed immunofluorescence staining and observed decreased immunoreactivity of both LAMP2a and HSC70, as well as reduced co‐localization of the two proteins, indicating impaired CMA complex assembly. In contrast, the expression of LAMP1, used as an internal control, remained unchanged (Figure 3E,F and Figure S3E). These results demonstrate that IFNγ/LPS co‐stimulation suppresses CMA activity at the post‐translational level, independent of transcriptional regulation.

**FIGURE 3:**
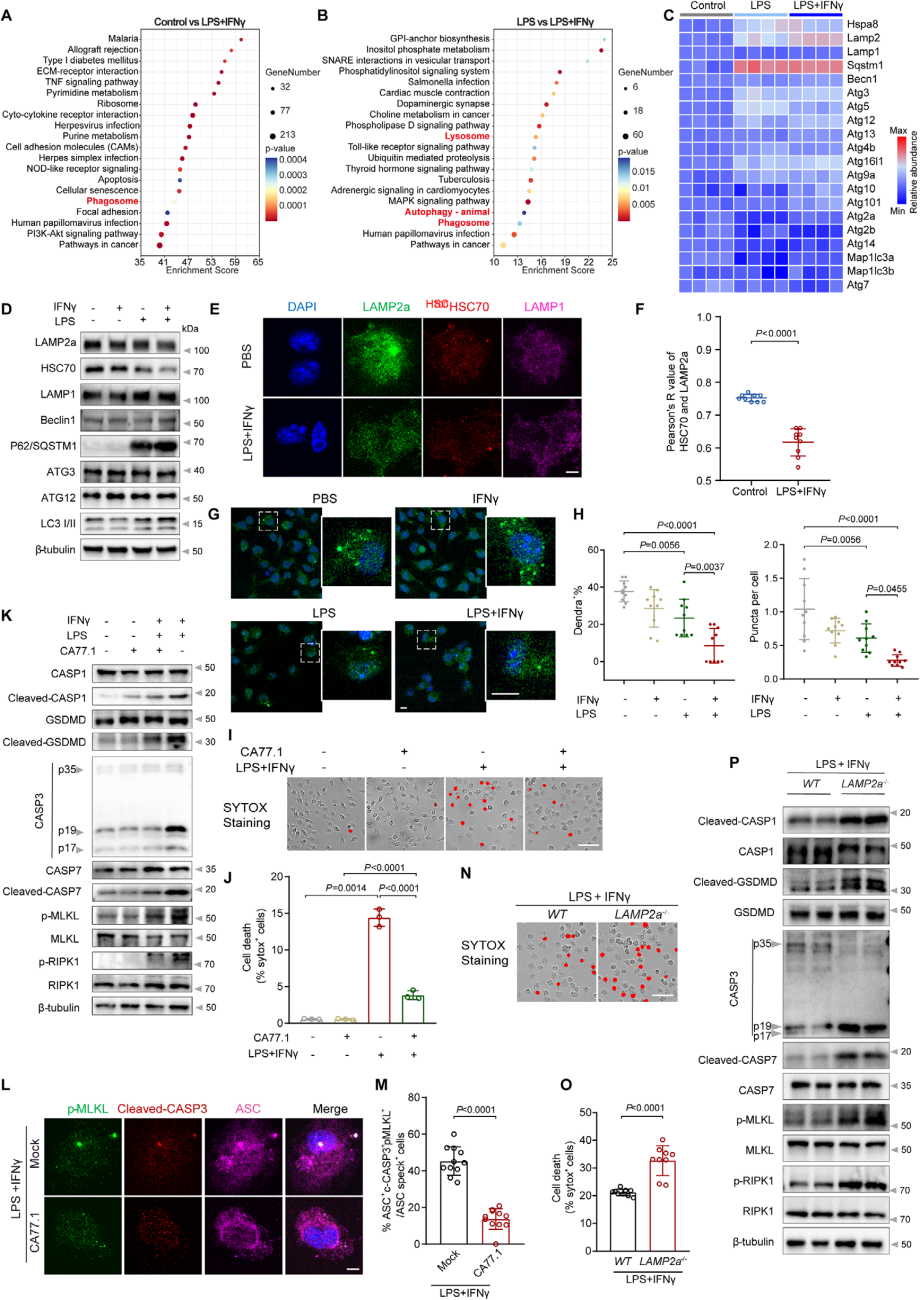
CMA downregulation upon IFNγ/LPS co‐stimulation induces PANoptosis in macrophages. (A,B) KEGG analysis of differentially expressed genes in RNAseq between PBS and LPS+IFNγ treated (A) and between LPS and LPS+IFNγ treated (B) BMDMs. (C) Heatmap depicting relative mRNA levels of genes involved in autophagy in BMDMs treated with PBS, LPS or LPS+IFNγ. (D) Immunoblotting analysis of protein expression related to autophagy in BMDMs treated with PBS, IFNγ, LPS or LPS+IFNγ. (E,F) Representative immunofluorescence images of endogenous LAMP2a, HSC70 and LAMP1 (E) and statistical analyses of co‐localization of LAMP2a and HSC70 (F, n = 9) in BMDMs with or without 24 h LPS+IFNγ stimulation. Scale bar = 5 µm. (G‐H) Representative immunofluorescence images of BMDMs extracted from KFERQ‐Dendra2 treated with PBS, IFNγ, LPS or LPS+IFNγ (G) and statistical analyses of percent of Dendra^+^ cell (Dendra^+^: puncta≥2 in one single cell, n = 10) and puncta per cell (n = 10). Scale bar = 10 µm. (I‐J) Percent of BMDMs death with the treatment of PBS, CA77.1, LPS+IFNγ or CA77.1‐ LPS+IFNγ (stimulation of LPS+IFNγ with CA77.1 pretreated) using SYTOX staining (n = 3). Scale bar = 50 µm. (K) Immunoblotting analysis of pro‐ and cleaved CASP1, pro‐ and cleaved GSDMD; pro‐ (P35) and cleaved (P17/19) CASP3, pro‐ and cleaved CASP7; pRIPK1 and tRIPK1, pMLKL and tMLKL in PBS, CA77.1, CA77.1‐ LPS+IFNγ or LPS+IFNγ treated BMDMs. (L,M) Representative immunofluorescence images of ASC, c‐CASP3, and pMLKL in BMDMs stimulated with LPS+IFNγ pretreated with or without CA77.1 (L). White dots indicate the ASC speck. Scale bar = 5 µm. Quantification of the percentage of cells with ASC^+^c‐CASP3^+^pMLKL^+^ specks among the ASC speck^+^ cells (M, n = 11). (N,O) Percent of *WT* and *LAMP2a^−/−^* BMDMs that are dead after stimulation with LPS+IFNγ using SYTOX staining (n = 9). Scale bar = 50 µm. (P) Immunoblotting analysis of pro‐ and cleaved CASP1, pro‐ and cleaved GSDMD; pro‐ and cleaved CASP3, pro‐ and cleaved CASP7; pRIPK1 and tRIPK1, pMLKL and tMLKL in LPS+IFNγ treated *WT* and *LAMP2a^−/−^* BMDMs. Data are representative of at least three independent experiments. Data are shown as mean ± SD. Analysis was performed using the two‐tailed Student's *t*‐tests (F, M and O) or one‐way ANOVA (H and J).

To further validate the downregulation of CMA, we employed KFERQ‐Dendra transgenic mice, a model that enables visualization of CMA activation via a shift in fluorescence distribution from a diffuse cytosolic pattern to distinct puncta. Consistent with our biochemical results, a significant reduction in both puncta per cell and the proportion of Dendra‐positive cells (defined as cells containing ≥2 puncta) was observed in IFNγ/LPS co‐stimulated BMDMs (Figure 3G,H), indicating markedly suppressed CMA activity in vitro. This suppression was further corroborated in vivo, where IFNγ/LPS co‐treatment challenge led to a notable decrease in the colocalization of F4/80‐positive macrophages and CMA puncta in lung, liver, and kidney tissues (Figure S3F–H). Collectively, these findings demonstrate that IFNγ/LPS co‐stimulation induced inflammatory storm robustly suppresses CMA function in macrophages both in vitro and in vivo.

To conclusively determine the role of CMA in regulating PANoptosis, we employed the CMA activator CA77.1. First of all, pretreatment with CA77.1 markedly reduced IFNγ/LPS co‐stimulation‐induced BMDM death and LDH release (Figure 3I,J and Figure S3J). Immunoblot analysis confirmed that IFNγ/LPS co‐stimulation‐induced PANoptosis events were reversed by CA77.1 treatment in BMDMs (Figure 3K). This reversion was further corroborated by immunofluorescence, which displayed a decreased formation of co‐localized ASC^+^c‐CASP3^+^pMLKL^+^ specks after CA77.1 pretreatment (Figure 3L,M). Interestingly, CA77.1 mitigated IFNγ/LPS co‐stimulation‐induced proinflammatory cytokine production and release in BMDMs at the same time (Figure S3L). As the initial trigger of BMDM death, we reasonably presumed that CMA activator rescued BMDMs from inflammatory storm‐induced death by intervening in the positive feedback of “inflammatory storm” and “macrophage death”. Subsequently, to further corroborate the specific role of CMA, we utilized *LAMP2a* ^−/ −^ models. Upon IFNγ/LPS co‐stimulation, *LAMP2a* ^−/ −^ BMDMs exhibited enhanced cell death and membrane damage (Figure 3N,O and Figure S3K), and elevated activity of key executors of PANoptosis compared to wild‐type (*WT*) cells (Figure 3P). It is noteworthy that a more severe cytokine storm (IL‐1β, IL‐6, TNF‐α) was observed in *LAMP2a* ^−/ −^ BMDMs (Figure S3M), demonstrating that deficient CMA could induce macrophage inflammation, consistent with previous work [23, 37]. Combined with the proven inflammatory storm‐induced CMA downregulation in BMDMs in this work, we established another vicious feedback between “inflammatory storm” and “CMA dysfunction”, which could also be blocked by CA77.1. Additionally, to exclude the contribution of macroautophagy, we performed siRNA knockdown of ATG5 and ATG7 in BMDMs. PANoptosis was comparable between siATG5/siATG7 and siNC groups (Figure S3D), indicating that macroautophagy does not contribute to PANoptosis in our model.

Collectively, these gain‐of‐function (CA77.1 activation) and loss‐of‐function (*LAMP2a* ^−/ −^) experiments provide complementary and conclusive evidence that CMA acts as a critical negative regulator of PANoptosis in macrophages during septic inflammatory stress, potentially by interrupting the vicious cycle involving proinflammatory cytokines, macrophage death, and subsequent cytokine release. Furthermore, our findings propose an additional regulatory circuit between dysfunctional CMA and inflammatory storm, offering an alternative explanation for the inverse correlation between CMA activity and inflammatory cytokine levels.

### 2.4 CMA Downregulation Promotes Pyrin‐Dependent Assembly of PANoptosome

PANoptosis is an inflammatory programmed cell death pathway mediated by the PANoptosome complex, which coordinates the assembly of sensors (AIM2, NLRP3, Pyrin, ZBP1), adaptors (ASC), and effector molecules (CASP1/3/7, MLKL) [16, 19]. To identify the key sensor protein responsible for initiating PANoptosome assembly in IFNγ/LPS co‐stimulation‐induced PANoptosis, we compared responses of BMDMs lacking various sensors (*AIM2* ^−/ −^, *NLRP3* ^−/ −^, *Pyrin* ^−/ −^, or *ZBP1* ^−/ −^). Among the sensor deficiencies screened, genetic ablation of *Pyrin* resulted in the most pronounced reduction in SYTOX‐positive cell death (Figure 4A,B). Additionally, cytokine profiling demonstrated the most effective suppression of inflammatory responses in *Pyrin* ^−/ −^ BMDMs (Figure S4I), reinforcing the functional importance of Pyrin in driving inflammatory outcomes. Consistent with this observation, *Pyrin* deficiency led to the most marked attenuation of ASC speck formation, a hallmark of inflammasome‐driven PANoptosome assembly, following IFNγ/LPS co‐stimulation (Figure 4C,D). Immunoblot analysis further confirmed the most significant attenuation of PANoptosis markers in *Pyrin* ^−/ −^ BMDMs, which was reversed by lentiviral Pyrin overexpression (Figure 4E and S4K), underscoring the central role of Pyrin in this cell death pathway.

**FIGURE 4:**
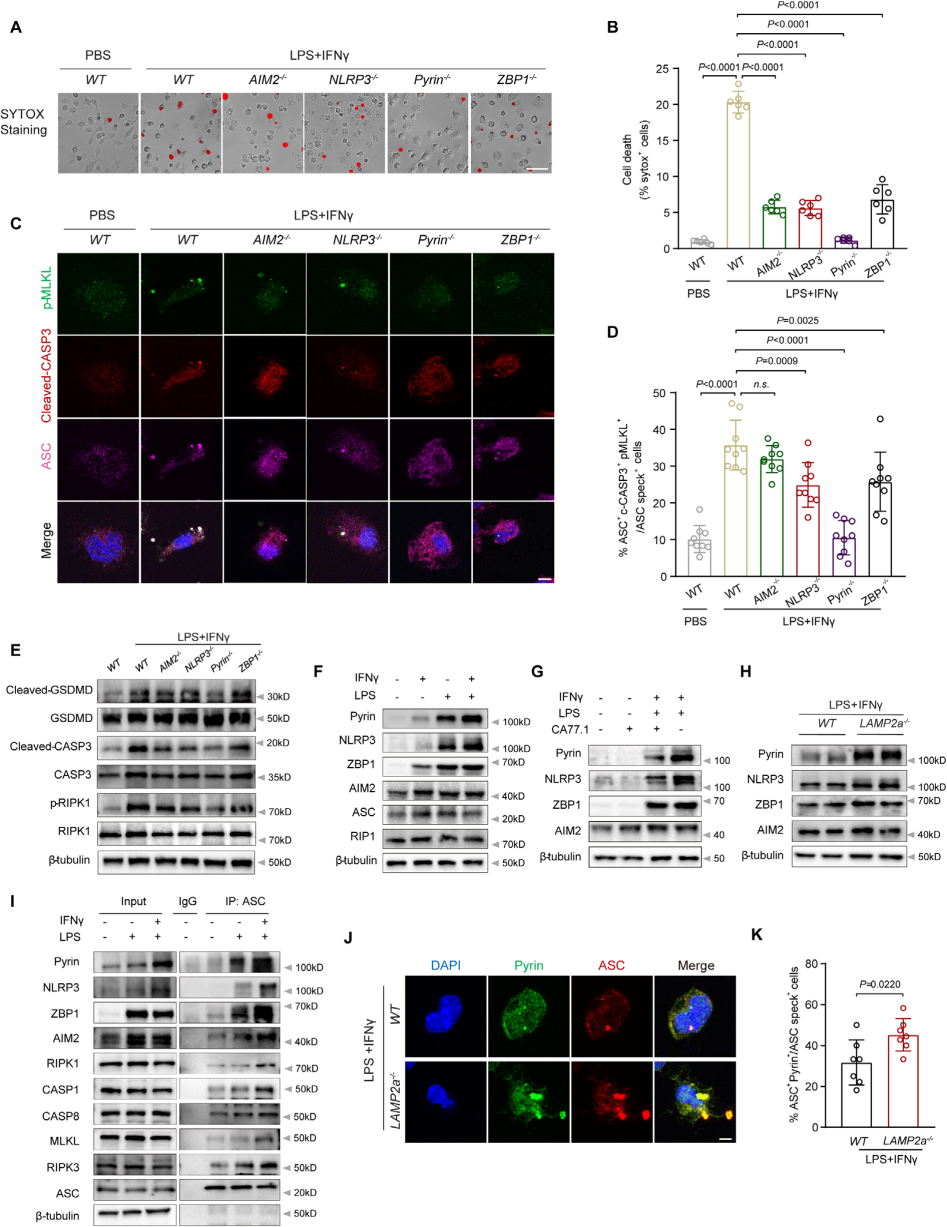
CMA dysfunction facilitates Pyrin expression and Pyrin‐dependent PANoptosome assembly. (A,B) Percent of death in *WT*, *AIM2^–/–^*, *NLRP3^–/–^*, *Pyrin^–/–^*, *ZBP1^–/–^* BMDMs stimulated with PBS or LPS+IFNγ (n = 6). Scale bar = 50 µm. (C) Representative immunofluorescence images of ASC, c‐CASP3, and pMLKL in *WT*, *AIM2^−/−^*, *NLRP3^−/−^*, *Pyrin^−/−^*, *ZBP1^−/−^* BMDMs 24 h after LPS and IFNγ stimulation. Scale bar = 5 µm. (D) Quantification of the percentage of cells with ASC^+^c‐CASP3^+^pMLKL^+^ specks among the ASC speck^+^ cells (n = 9). (E) Immunoblotting analysis of pro‐ and cleaved GSDMD, pro‐ and cleaved CASP3, pRIPK1, and tRIPK1 in unstimulated and LPS+IFNγ‐stimulated *WT*, *AIM2^−/−^*, *NLRP3^−/−^*, *Pyrin^−/−^*, *ZBP1^−/−^* BMDMs. (F) Immunoblotting analysis of AIM2, NLRP3, Pyrin, ZBP1, ASC, and PK1 in BMDMs treated with PBS, IFNγ, LPS or LPS+IFNγ. (G) Immunoblotting analysis of AIM2, NLRP3, Pyrin, and ZBP1 in BMDMs treated with PBS, CA77.1, LPS+IFNγ or CA77.1‐ LPS+IFNγ. (H) Immunoblotting analysis of AIM2, NLRP3, Pyrin, and ZBP1 in *WT* and *LAMP2a^−/−^* BMDMs treated with LPS+IFNγ. (I) Immunoprecipitation analysis for the assembly of PANoptosome and immunoblot analysis for ASC, AIM2, NLRP3, Pyrin, ZBP1, RIPK1, RIPK3, CASP1, CASP8 and MLKL in BMDMs treated with PBS, LPS or LPS+IFNγ. (J‐K) Representative immunofluorescence images of ASC and Pyrin in *WT* and *LAMP2a^−/−^* BMDMs 24 h after LPS and IFNγ stimulation (J) and quantification of the percentage of cells with ASC^+^Pyrin^+^ specks among the ASC speck^+^ cells (K, n = 7). Scale bar = 5 µm. Data are representative of at least three independent experiments. Data are shown as mean ± SD. Analysis was performed using the two‐tailed Student's *t*‐test (K) or one‐way ANOVA (B and D). *n.s*. stands for not significant.

Mechanistically, co‐immunoprecipitation (Co‐IP) assays revealed enhanced interaction between ASC and the sensor proteins, including AIM2, NLRP3, Pyrin, and ZBP1, in BMDMs treated with IFNγ/LPS compared to LPS alone (Figure 4I). Specifically, the augmented PANoptosome assembly coincided with a selective elevation in the protein expression of Pyrin and NLRP3, while AIM2 and ZBP1 levels remained unchanged upon IFNγ/LPS co‐stimulation (Figure 4F). Given that NLRP3 is a well‐characterized CMA substrate, we therefore prioritized elucidating the role of Pyrin. Consistent with the protein results, qPCR analysis revealed a specific upregulation of *Pyrin* mRNA, with no significant changes in other PANoptosome components (Figure S4A–D). RNA‐seq analysis also demonstrated consistency of Pyrin expression pattern across the three groups with qPCR (Figure S4J).

To investigate whether CMA regulates Pyrin‐mediated PANoptosis, we assessed Pyrin expression under CMA‐modulating conditions. Immunoblot and qPCR analyses demonstrated that pharmacological activation of CMA downregulated Pyrin expression at both protein and mRNA levels (Figure 4G and Figure S4E), whereas genetic ablation of *LAMP2a* resulted in marked upregulation of Pyrin (Figure 4H and Figure S4G), compared to other sensors such as AIM2 (Figure 4G,H and Figure S4F,H). These findings indicate that Pyrin, but not other PANoptosome sensors, is specifically modulated by CMA in BMDMs treated with LPS plus IFNγ. Furthermore, immunofluorescence analysis confirmed enhanced colocalization of Pyrin with ASC in *LAMP2a*‐deficient BMDMs compared with *WT* following IFNγ/LPS co‐stimulation (Figure 4J,K). Collectively, these results establish that CMA negatively regulates Pyrin expression and thereby suppresses PANoptosis activation in macrophages.

### 2.5 Defective CMA Activity Drives CUX1 Accumulation to Enhance Pyrin Expression

The coordinated changes in Pyrin protein and mRNA levels upon IFNγ/LPS co‐treatment or under modulated CMA activity suggested that Pyrin is unlikely a direct substrate of CMA, but rather indirectly regulated through a transcription factor (TF) controlled by CMA. To identify this TF, we performed immunoprecipitation‐mass spectrometry (IP‐MS) using anti‐LAMP2a and anti‐HSC70 antibodies, which identified 7 candidate TFs (Figure 5A and Figure S5D). Among these, cut‐like homeobox 1 (CUX1) emerged as the most promising candidate based on its IP‐MS score and intensity, number of KFERQ motifs (Figure S5A,B,D), predicted binding site within the *Pyrin* promoter (Figure S5C), and positive correlation with *MEFV* expression in the GEPIA database (http://gepia.cancer‐pku.cn/) (Figure 5B).

**FIGURE 5:**
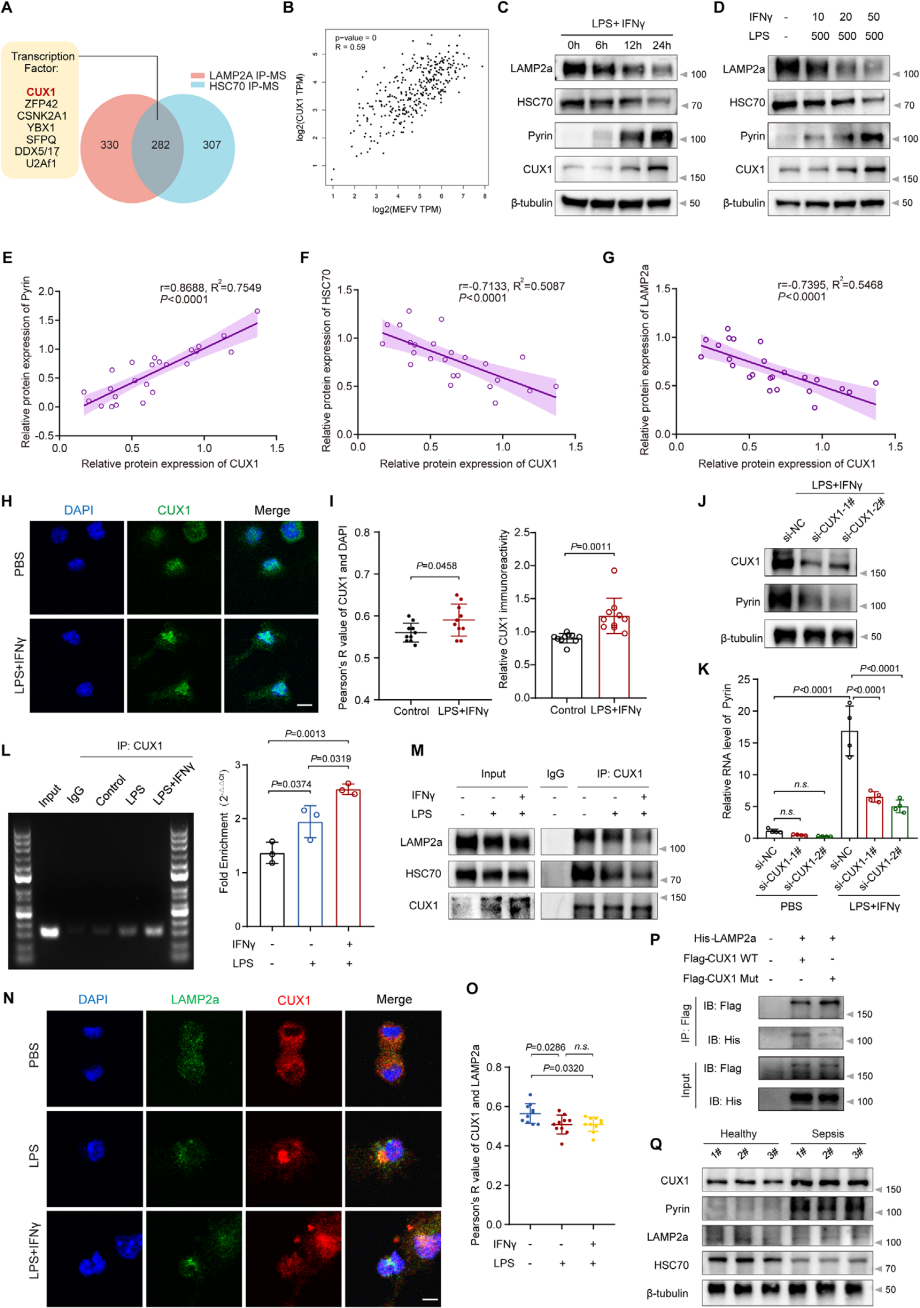
Defective CMA activity drives CUX1 accumulation to enhance Pyrin expression. (A) Venn diagram depicting the overlapping transcription factors found by IP‐MS produced by anti‐LAMP2a and anti‐HSC70 in BMDMs. (B) The correlation analysis of CUX1 and Mediterranean fever (*MEFV*, namely, Pyrin) in whole blood generated by the GEPIA database (http://gepia.cancer‐pku.cn/). (C,D) Immunoblotting analysis of LAMP2a, HSC70, Pyrin, and CUX1 in BMDMs treated with LPS+IFNγ at the indicated concentration for 24 h and with the indicated time with 500 ng/mL of LPS and 50 ng/mL of IFNγ. (E–G) Correlation analysis between the protein expression of CUX1 and Pyrin (E), HSC70 (F), and LAMP2a (G) from immunoblotting analysis (n = 24). (H,I) Representative immunofluorescence images of CUX1 and DAPI in BMDMs treated with or without LPS+IFNγ (H). Co‐localization analysis of CUX1 and DAPI by means of Pearson's R value and relative CUX1 immunoreactivity analysis in PBS‐ or LPS+IFNγ‐treated BMDMs (I, n = 10). Scale bar = 10 µm. (J) Immunoblotting analysis of CUX1 and Pyrin in BMDMs transfected with control siRNA or *CUX1* siRNAs. (K) Relative mRNA expression of *Pyrin* in BMDMs transfected with control siRNA or *CUX1* siRNAs stimulated with or without LPS+IFNγ (n = 4). (L) Chromatin immunoprecipitation (ChIP) assay for detection of CUX1 binding to the promoter of *Pyrin* in BMDMs treated with PBS, LPS, or LPS+IFNγ and quantification (n = 3). Normal IgG was used as a negative control. Fold enrichment was normalized to input and calculated by the 2^−ΔΔCt^ method relative to IgG. (M) Co‐immunoprecipitation of CUX1 with LAMP2a and HSC70 from BMDMs treated with PBS, LPS, or LPS+IFNγ. (N,O) Representative immunofluorescence images of CUX1 and LAMP2a in BMDMs treated with PBS, LPS, or LPS+IFNγ (N) and co‐localization analysis of the two (O, n = 10). Scale bar = 5 µm. (P) Co‐immunoprecipitation of His‐LAMP2a with wild‐type (WT) Flag‐CUX1 (Flag‐CUX1 WT) or mutant Flag‐CUX1 (Flag‐CUX1 Mut) from HEK293T cells. All of the eight KFERQ motifs in mouse CUX1 were mutated to disrupt the interaction of CUX1 with the CMA receptor LAMP2a. (Q) Immunoblotting analysis of CUX1, Pyrin, LAMP2a, and HSC70 in PBMCs from sepsis and healthy patients. Data are representative of at least three independent experiments. Data are shown as mean ± SD. Analysis was performed using the two‐tailed Student's *t*‐tests (I), one‐way ANOVA (K, L and O), or Pearson's correlation (E–G).

Although CUX1 has established roles in neuronal differentiation and cellular maturation across various tissues [38, 39], its potential involvement in regulating inflammasome expression remained unexplored. We intended to validate whether CUX1 is necessary for IFNγ/LPS co‐stimulation‐induced *Pyrin* transcription. First, we observed a synchronized, dose‐ and time‐dependent accumulation of CUX1 and Pyrin in BMDMs following IFNγ/LPS co‐stimulation challenge, which occurred concomitantly with the downregulation of the CMA components, LAMP2a and HSC70 (Figure 5C,D). Moreover, the correlation analysis showed a significant negative relation between CUX1 and LAMP2a/HSC70, and a positive relation between CUX1 and Pyrin protein expression (Figure 5E–G). Besides, immunofluorescence analysis revealed a pronounced increase in the colocalization of CUX1 with DAPI and CUX1 immunoreactivity following IFNγ/LPS co‐treatment (Figure 5H,I), indicating stimulus‐induced nuclear translocation of CUX1 and suggesting its enhanced transcriptional activation. Next, genetic silencing of *CUX1* (Figure S5G) resulted in a marked downregulation of Pyrin at both the protein and mRNA levels (Figure 5J,K). Finally, we verified and quantified the binding of CUX1 to the promoter of *Pyrin*, which could be enhanced by co‐treatment with LPS plus IFNγ (Figure 5L). These findings uncover CUX1 as a novel transcriptional regulator of Pyrin inflammasome expression.

Next, we sought to determine whether CUX1 serves as a CMA substrate. A CMA substrate must contain KFERQ motifs in its amino acid sequence. We attested to the existence of the motifs in CUX1 from KFERQ‐finder V0.8 (https://rshine.einsteinmed.edu/) (Figure S5M,N). A negative relation between CUX1 and LAMP2a/HSC70 protein expression was confirmed by correlation analysis (Figure 5F,G). Intriguingly, the mRNA expression of CUX1 was even lower after IFNγ/LPS co‐treatment (Figure S5E,F), despite the reinforcement of protein expression (Figure 5C,D). Similarly, in *LAMP2a^−/−^* BMDMs, CUX1 protein, but not its mRNA, was elevated (Figure S5H,I), supporting the premise that CUX1 is subject to post‐translational regulation via CMA. Additionally, CA77.1 pretreatment mitigated accumulation of CUX1(Figure S9C). To figure out the reason for the opposite trends of protein and mRNA expression of CUX1 upon IFNγ/LPS co‐stimulation, we applied 3‐Methyladenine (3‐MA), chloroquine (CQ), MG‐132, and ammonium chloride (NH_4_Cl) to inhibit key degradation pathways. Pretreatment with lysosomal inhibitors (CQ and NH_4_Cl), but not proteasomal or autophagic inhibitors, led to marked accumulation of CUX1 (Figure S5J), indicating that CUX1 degradation is predominantly lysosome‐dependent. Cycloheximide (CHX) chase assay indicated that LPS+IFNγ stimulation significantly prolonged the half‐life of CUX1 compared with the Control group, consistent with impaired CMA‐mediated degradation (Figure S9A). Following treatment with the CMA activator CA77.1, CUX1 protein levels were markedly reduced at 6 h after CHX administration (Figure S9B). In contrast, in LAMP2a‐deficient cells, CUX1 protein levels remained elevated at 6 h after CHX administration (Figure S9D).

Next, we assessed the physical interaction between CUX1 and the CMA machinery. Co‐immunoprecipitation assays confirmed the binding of CUX1 to LAMP2a and HSC70, and the interactions were significantly attenuated under IFNγ/LPS co‐stimulation (Figure 5M and Figure S5K,L). Immunofluorescence analysis further demonstrated colocalization of CUX1 with LAMP2a, which was likewise diminished by IFNγ/LPS co‐treatment (Figure 5N,O). To conclusively validate CUX1 as a CMA substrate, we mutated eight putative KFERQ‐like motifs in CUX1 (Figure S5O). This mutant exhibited significantly reduced binding to LAMP2a compared to the WT in co‐transfection experiments (Figure 5P). CHX chase assay also demonstrated that mutation of the KFERQ‐like motif in CUX1 stabilized the protein (Figure S9G), confirming that CUX1 degradation is mediated through the CMA recognition motif.

Hence, we validate a “CMA‐CUX1‐Pyrin” axis involved in regulating PANoptosis during septic inflammation storm. Subsequent analysis of key proteins in this axis revealed decreased protein levels of LAMP2a and HSC70, accompanied by accumulation of CUX1 and its downstream target Pyrin in septic patients (Figure 5Q). To further validate this axis, we performed CUX1 knockdown in both *WT* and *LAMP2a* ^−/ −^ BMDMs using siCUX1. Indeed, CUX1 knockdown significantly reduced PANoptosis (Figure S10A), and in *LAMP2a* ^−/ −^ BMDMs, CUX1 knockdown also suppressed PANoptosis (Figure S10B). This HSC70 lactylation‐CMA‐CUX1‐Pyrin axis was also validated in the CLP model. We performed ex vivo analyses in lung and liver tissues harvested from CLP vs. sham mice. CUX1, Pyrin, and PANoptosis markers (Figure S8A,B), and HSC70 lactylation (Figure S8C,D) were upregulated in the lung and liver of CLP mice compared with sham mice. CMA activity was impaired in CLP mice, as evidenced by decreased LAMP2a and HSC70 protein levels in lung and liver tissues (Figure S8A,B) and lower Dendra fluorescence in the liver, lung, and kidney of CLP mice (Figure S8E,F). Collectively, these results indicate that under septic inflammatory conditions, CMA dysfunction, characterized by impaired substrate‐receptor interaction, leads to the stabilization of the transcription factor CUX1. This accumulation drives the upregulation of Pyrin, which ultimately promotes inflammasome assembly and macrophage PANoptosis.

### 2.6 HSC70 Lactylation at K561 Mediates CMA Dysfunction in Septic Inflammatory Storm

Next, we explored the mechanism underlying CMA dysregulation during septic cytokine storm. RNA sequencing revealed a metabolic shift from oxidative phosphorylation (OXPHOS) to glycolysis in BMDMs treated with LPS plus IFNγ (Figure 6A and S6A). In line with this, Seahorse assay measurements of oxygen consumption rate (OCR) and extracellular acidification rate (ECAR) exhibited similar results. Both basal and compensatory glycolysis were remarkably elevated in BMDMs stimulated with LPS, which were further enhanced by IFNγ/LPS co‐treatment (Figure 6B). In contrast, mitochondrial metabolism was inhibited as evidenced by reduced basal and maximal respiration following LPS stimulation, which was further diminished upon IFNγ/LPS exposure (Figure S6B). Given the enhanced glycolytic flux during septic inflammatory storm, our analysis revealed a time‐dependent accumulation of lactate, with the most pronounced elevation observed in the serum and supernatants of the IFNγ/LPS co‐stimulated group compared to the PBS, IFNγ, or LPS alone treatments (Figure 6C and S6C,D). Meanwhile, immunoblot analysis also confirmed an increased PAN‐specific lysine lactylation (Pan‐Kla) in vitro in BMDMs co‐stimulated with IFNγ/LPS (Figure 6F). Similar lactate accumulation was also observed in septic patients (Figure 6D), accompanied by a markedly upregulated Pan‐Kla modification in their PBMCs (Figure 6E). Of note, their lactate levels showed a significant negative correlation with their macrophage count (Figure 6G).

**FIGURE 6:**
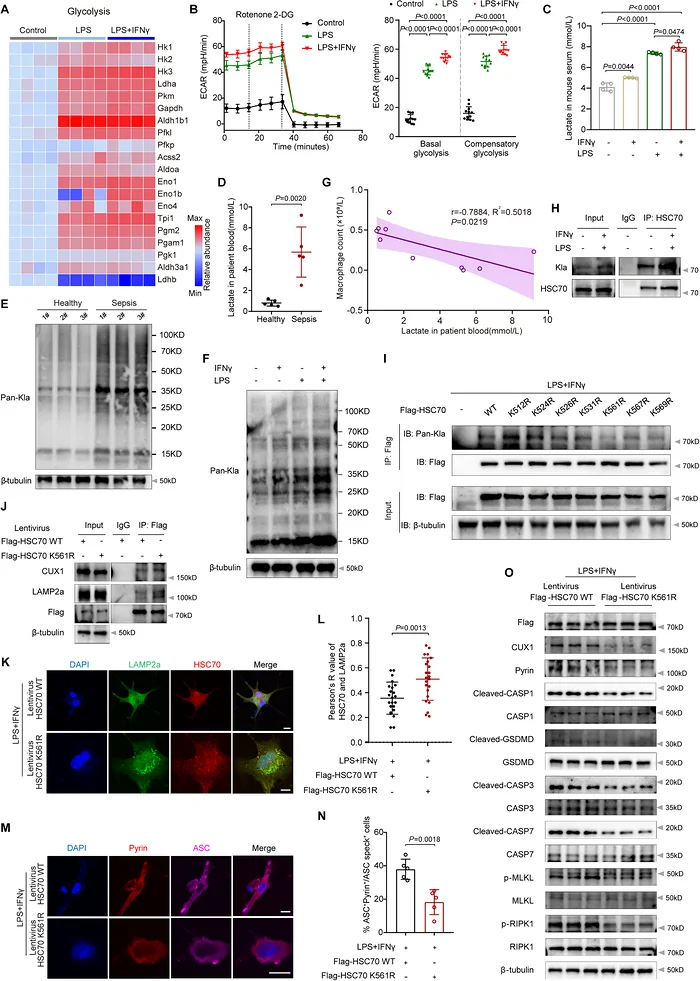
Lactylation of HSC70 at site K561 mediates CMA defection induced by IFNγ/LPS co‐stimulation. (A) Heatmap depicting relative mRNA levels of genes involved in the pathway of “Glycolysis / Gluconeogenesis” in BMDMs treated with PBS, LPS, or LPS+IFNγ. (B) Extracellular acidification rate (ECAR, B (left)) assessed by Seahorse Assay in BMDMs treated with PBS, LPS, or LPS+IFNγ, and quantification of basal and compensatory glycolysis (B (right)) (n = 12). (C) Serum lactate levels in mice injected with PBS, IFNγ, LPS, or LPS+IFNγ detected by a lactate assay kit. (n = 4) (D) Lactate levels in the blood of sepsis and healthy patients (n = 5). (E) Immunoblotting analysis of PAN‐Kla in PBMCs from sepsis and healthy patients. (F) Immunoblotting analysis of PAN‐specific lysine lactylation (PAN‐Kla) in BMDMs treated with PBS, IFNγ, LPS, or LPS+IFNγ. (G) Correlation analysis between the level of lactate and the macrophage count in the blood of healthy and sepsis patients (n = 10). (H) Co‐immunoprecipitation of HSC70 with lysine lactylation (Kla) from BMDMs treated with or without LPS+IFNγ for confirmation of lactylation of HSC70 upon co‐stimulation with LPS+IFNγ. (I) Co‐immunoprecipitation of Flag with PAN‐Kla from HEK293T cells stimulated with LPS+IFNγ and transfected with Flag‐HSC70 WT and the other seven mutated Flag‐HSC70 plasmids with replacement of potentially lactylated lysine (K) with arginine (R) for abolishing lactylation. (J) BMDMs transfected with *HSC70* siRNA and reconstituted with Flag‐tagged HSC70‐WT or HSC70‐K561R via lentiviral infection were immunoprecipitated by anti‐Flag and immunoblotted for CUX1 and LAMP2a. (K,L) Representative immunofluorescence images of LAMP2a and HSC70 in BMDMs with treatment of LPS+IFNγ after transfected with Flag‐HSC70 WT or Flag‐HSC70 K561R lentivirus (K), and co‐localization analysis of LAMP2a with HSC70 by means of Pearson's R value (L, n = 23). Scale bar = 10 µm. (M‐N) Representative immunofluorescence images of ASC and Pyrin in BMDMs with treatment of LPS+IFNγ after transfected with Flag‐HSC70 WT or Flag‐HSC70 K561R lentivirus (M), and quantification of the percentage of cells with ASC^+^Pyrin^+^ specks among the ASC speck^+^ cells (N, n = 5). Scale bar = 10 µm. (O) Immunoblotting analysis of pro‐ and cleaved CASP1, pro‐ and cleaved GSDMD; pro‐ and cleaved CASP3, pro‐ and cleaved CASP7; pRIPK1 and tRIPK1, pMLKL and tMLKL, CUX1, Pyrin and Flag in LPS+IFNγ‐treated Flag‐HSC70 WT or Flag‐HSC70 K561R lentivirally transfected BMDMs. Data are representative of at least three independent experiments. Data are shown as mean ± SD. Analysis was performed using the two‐tailed Student's *t*‐tests (D, L and N), one‐way ANOVA (B,C) or Pearson's correlation (G).

To investigate whether and how lactylation impacts CMA activity and macrophage fate, we performed IP‐MS and identified 663 proteins lactylated in BMDMs upon LPS plus IFNγ stimulation, among which HSC70 exhibited relatively high modification intensity (Figure S6E,F). Co‐IP validated HSC70 lactylation (Figure 6H), leading us to hypothesize that HSC70 lactylation mediates CMA impairment in sepsis‐associated cytokine storm. Subsequently, anti‐HSC70 IP‐MS analysis showed seven potential sites for lactylation in HSC70 (Figure S6G,H). Accordingly, we generated these mutant plasmids by replacing lysine (K) with arginine (R) to abrogate lactylation. Transfection of HEK293T cells with WT or mutant Flag‐HSC70 constructs, followed by Co‐IP, identified K561 as the principal lactylation site (Figure 6I). Functional analysis showed that compared to WT Flag‐HSC70, the K561R mutant enhanced LAMP2a‐HSC70 colocalization (Figure S6I,J) and their interaction (Figure S6L), indicating restored CMA activity. Consequently, CMA‐mediated degradation of the transcription factor CUX1 was recovered, as demonstrated by strengthened HSC70‐CUX1 interaction (Figure S6K) and reduced CUX1 expression in K561R‐transfected HEK293T cells (Figure S6M). Furthermore, we validated the key role of lactylation of HSC70 at K561 in primary macrophages by reconstitution with WT and K561R HSC70 lentivirus. Similar results were derived from BMDMs. We found reconstitution with HSC70‐K561R enhanced LAMP2a‐HSC70 co‐localization compared with HSC70‐WT (Figure 6K,L). Co‐immunoprecipitation of HSC70 showed enhanced binding to both LAMP2a and CUX1 in K561R‐reconstituted BMDMs (Figure 6J). K561R reconstitution also reduced ASC^+^Pyrin^+^ speck formation, CUX1 and Pyrin expression, as well as PANoptosis marker levels compared with HSC70‐WT (Figure 6M–O). The corresponding molecular docking and molecular dynamics simulation results of HSC70 and CUX1 are shown in Figure S15. K561 lactylation was associated with altered local dynamics (mean Cα RMSF of residues 555–570: 1.31 → 1.10 Å; lid‐SBDβ distance: 64.94 → 62.32 Å, both consistent across five 20‐ns blocks) and with interface redistribution at the CUX1‐binding surface (HSC70 610‐CUX1 occupancy: 98.0% → 9.3%; HSC70 561–610 intramolecular occupancy: 7.5% → 97.6%; contacting HSC70 residues: 62 → 53). Together, these results suggest a possible structural mechanism in which K561 lactylation changes local conformational constraints and reorganizes substrate engagement. These single‐trajectory simulations of a predicted, nucleotide‐free fragment complex provide evidence for conformational and contact changes, but do not independently quantify binding affinity.

As for the specific underlying mechanism for CMA dysfunction, by adopting CHX and MG132, 3‐MA, and CQ, and NH_4_Cl pretreatment, we confirmed that both LAMP2a and HSC70 degradation were primarily dependent on the proteasome pathway upon IFNγ/LPS stimulation (Figure S12A). The degradation probably accounts for the downregulation of LAMP2a and HSC70 in this condition. Subsequently, we verified that IFNγ/LPS stimulation markedly enhanced HSC70 ubiquitination, as detected by HSC70 immunoprecipitation followed by ubiquitin immunoblotting (Figure S12B), which could be reversed by the K561R mutation (Figure S12C). CHX chase assays yielded results consistent with the above findings. Under LPS+IFNγ stimulation, the half‐life of HSC70 was significantly shortened compared with the Control group (Figure S9A). In 293T cells, ectopic expression of the K561R mutant led to markedly delayed HSC70 degradation compared with wild‐type HSC70 (Figure S9F). These data indicate that K561 lactylation promotes HSC70 protein degradation and reduces its half‐life.

Together, these results confirm that lactylation of HSC70 at K561 promotes HSC70 protein degradation and reduces its half‐life, probably underlying IFNγ/LPS co‐stimulation‐induced CMA dysfunction.

### 2.7 Abrogation of HSC70 Lactylation Restores CMA Activity and Attenuates Macrophage PANoptosis

In our study, we employed oxamate to block lactate production. Pretreatment with oxamate significantly reduced the levels of IL‐1β, IL‐6, and TNF‐α, indicating attenuation of the inflammatory storm (Figure S7A). Furthermore, in KFERQ‐Dendra BMDMs co‐stimulated with IFNγ/LPS, oxamate pretreatment led to an increase in puncta formation (Figure 7A–C), indicating that lactate blockade effectively restored CMA activity in macrophages. Consistent with these findings, blocking lactate diminished the proportion of SYTOX‐positive macrophages (Figure 7D,E) and reduced membrane damage, as evidenced by the LDH assay (Figure 7F). Immunoblot analysis confirmed that the oxamate pretreatment reversed the inflammatory storm‐induced PANoptosis (Figure 7G), which was further corroborated by a significant reduction in the colocalization of ASC^+^c‐CASP3^+^pMLKL^+^ specks (Figure 7H,I).

**FIGURE 7:**
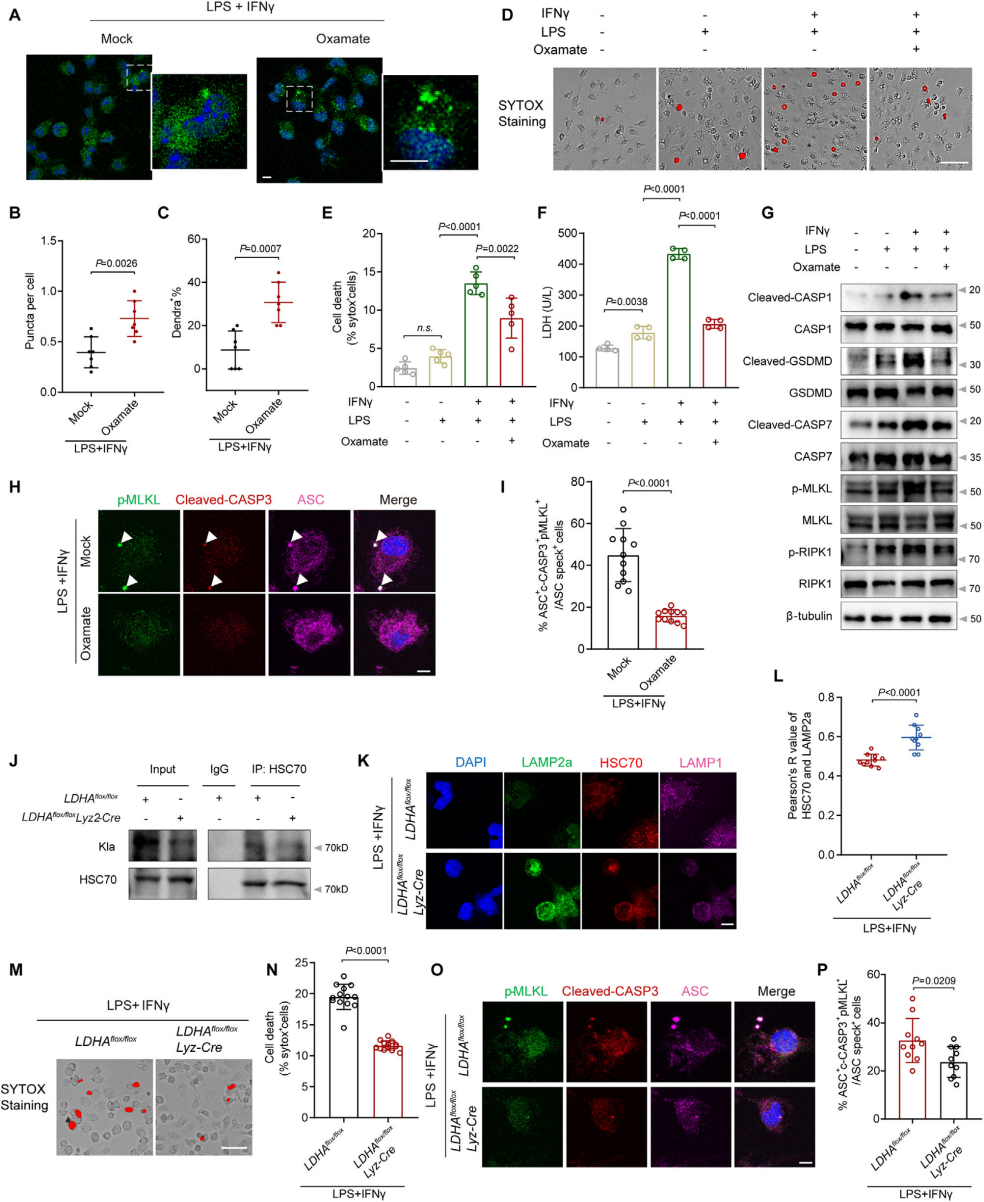
Blocking the lactylation of HSC70 restores CMA activity and alleviates PANoptosis. (A–C) Representative immunofluorescence images of BMDMs extracted from KFERQ‐Dendra2 mice stimulated by LPS+IFNγ with or without oxamate pretreatment (A) and statistical analyses of puncta per cell (B, n = 7) and percent of Dendra^+^ cells (C, n = 7). Scale bar = 10 µm. (D,E) Percent of BMDM death after treatment with PBS, LPS, LPS+IFNγ, or oxamate‐treated LPS+IFNγ (stimulation of LPS+IFNγ with oxamate pretreated) using SYTOX staining (n = 5). Scale bar = 50 µm. (F) LDH release into supernatants of BMDMs treated with PBS, LPS, LPS+IFNγ, or oxamate‐treated LPS+IFNγ (n = 4). (G) Immunoblotting analysis of pro‐ and cleaved CASP1, pro‐ and cleaved GSDMD; pro‐ and cleaved CASP7; pRIPK1 and tRIPK1, pMLKL and tMLKL in PBS‐, LPS‐, LPS+IFNγ, or oxamate‐treated LPS+IFNγ BMDMs. (H,I) Representative immunofluorescence images of ASC, c‐CASP3, and pMLKL in BMDMs 24 h after LPS and IFNγ stimulation with or without oxamate pretreatment (H) and quantification of the percentage of cells with ASC^+^c‐CASP3^+^pMLKL^+^ specks among the ASC speck^+^ cells (I n = 11). White arrowheads indicate ASC speck. Scale bar = 5 µm. (J) Co‐immunoprecipitation of HSC70 with Kla from *LDHA^flox/flox^* and *LDHA^flox/flox^ Lyz2‐Cre* BMDMs with or without LPS+IFNγ stimulation for validation of diminished expression of HSC70‐Kla in *LDHA^flox/flox^ Lyz2‐Cre* BMDMs. (K,L) Representative immunofluorescence images of endogenous LAMP2a, HSC70, and LAMP1 (K) and statistical analyses of co‐localization of LAMP2a and HSC70 (L, n = 10) with 24 h LPS+IFNγ stimulation in *LDHA^flox/flox^* and *LDHA^flox/flox^ Lyz2‐Cre* BMDMs. Scale bar = 5 µm. (M,N) Percent of death after treatment with LPS+IFNγ in *LDHA^flox/flox^* and *LDHA^flox/flox^ Lyz2‐Cre* BMDMs using SYTOX staining (n = 13). Scale bar = 50 µm. (O,P) Representative immunofluorescence images of ASC, c‐CASP3, and pMLKL in *LDHA^flox/flox^* and *LDHA^flox/flox^ Lyz2‐Cre* BMDMs 24 h after LPS and IFNγ stimulation (O) and quantification of the percentage of cells with ASC^+^c‐CASP3^+^pMLKL^+^ specks among the ASC speck^+^ cells (P, n = 10). Scale bar = 5 µm. Data are representative of at least three independent experiments. Data are shown as mean ± SD. Analysis was performed using the two‐tailed Student's *t*‐tests (B‐C, I, L, N and P) or one‐way ANOVA (E‐F). *n.s*. stands for not significant.

To genetically validate the role of lactylation, we crossed *LDHA^flox/flox^* mice with *Lyz2‐Cre* mice to achieve macrophage‐specific *LDHA* knockout mice. BMDMs from *LDHA^flox/flox^ Lyz2‐Cre* mice exhibited markedly reduced HSC70 lactylation compared to controls (Figure 7J). The reduced HSC70 lactylation restored CMA activity, as shown by increased immunoreactivity of LAMP2a and HSC70, unaltered LAMP1 expression as controls, and facilitated colocalization of LAMP2a and HSC70 in *LDHA^flox/flox^ Lyz2‐Cre* BMDMs compared with *LDHA^flox/flox^* BMDMs (Figure 7K,L and Figure S7B), along with recovered protein levels of LAMP2a and HSC70 (Figure S7C). Consequently, macrophage‐specific deletion of LDHA led to reduced cell death and attenuated PANoptosis, evidenced by a significant decrease in SYTOX^+^ cells and ASC^+^c‐CASP3^+^pMLKL^+^ specks (Figure 7M–P). In summary, both pharmacological inhibition of lactate production with oxamate and genetic ablation of *LDHA* in macrophages demonstrated that blocking HSC70 lactylation alleviates cytokine storm, restores CMA function, and suppresses macrophage PANoptosis.

### 2.8 Targeting CMA or Lactylation Modulates Multi‐Organ Dysfunction in Septic Mice

Having established that CMA and lactylation regulate the inflammatory storm and macrophage PANoptosis in vitro, we sought to corroborate their effects in vivo. Consistent with the in vitro findings, intraperitoneal pretreatment with CA77.1 in mice markedly reduced serum levels of proinflammatory cytokines (IL‐1β, IL‐6, TNF‐α) and attenuated IFNγ/LPS induced PBMCs death (Figure 8A–C). The attenuation of macrophage death led to ameliorated organ pathological damage, improved organ function, and ultimately a significantly elevated survival rate (Figure 8D–G). Conversely, *LAMP2a^−/−^* mice challenged with IFNγ/LPS exhibited higher serum proinflammatory cytokine levels, exacerbated multi‐organ damage as assessed by histopathology and serum bioindicators (ALT, AST, Cr, and BUN), and a significantly lower survival rate compared to their wild‐type counterparts (Figure 8H–L).

**FIGURE 8:**
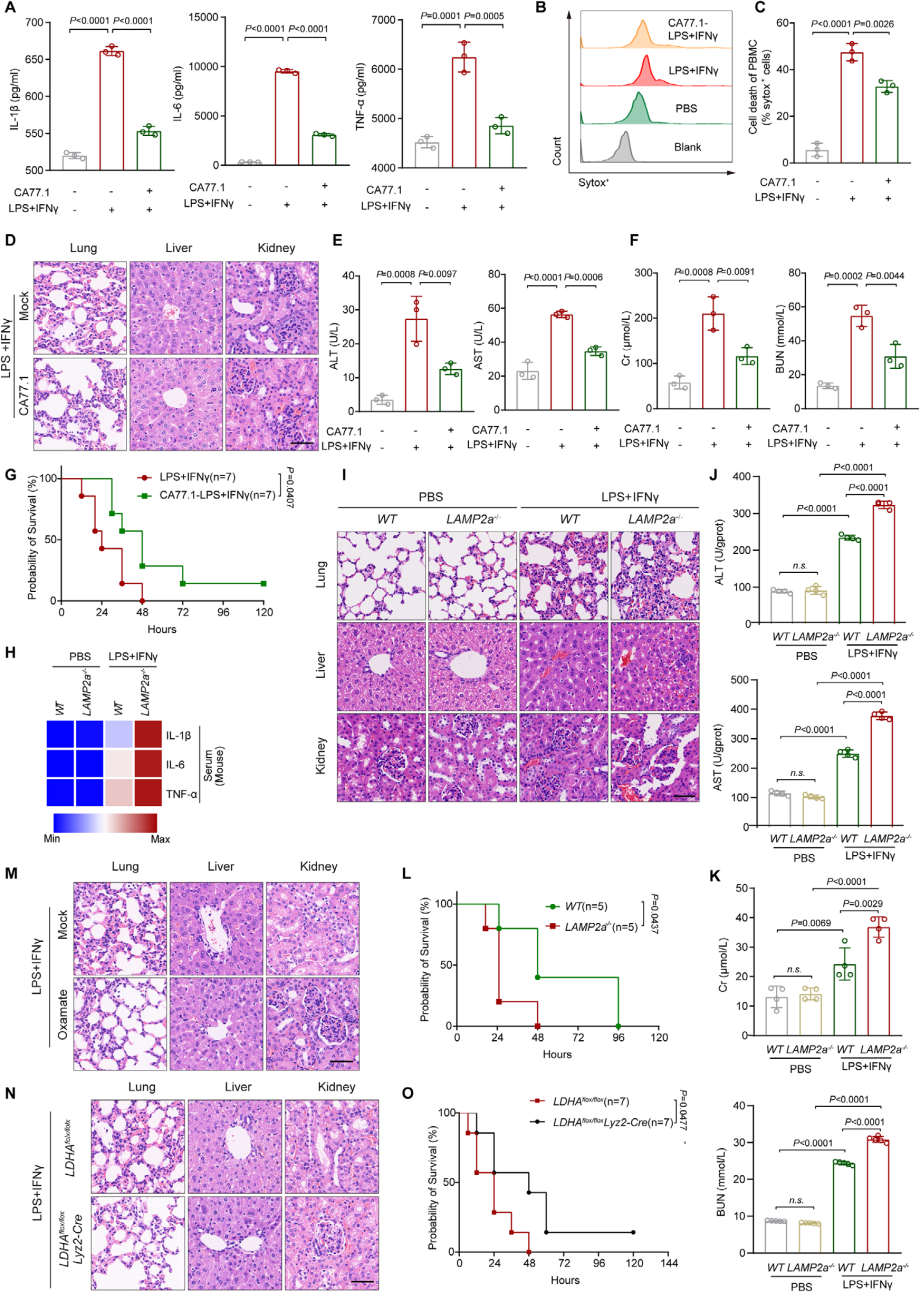
Intervention in CMA activity or lactylation modification affects multiple organ dysfunction in sepsis. (A) ELISA assay of murine serum IL‐1β, IL‐6, and TNF‐α 24 h after PBS, LPS+IFNγ, or CA77.1‐ LPS+IFNγ injection (n = 3). (B,C) Percent of PBMCs that are dead in mice injected with PBS, or LPS+IFNγ with or without CA77.1 pretreatment, using SYTOX staining detected by flow cytometer (n = 3). (D) Representative images of lung, liver, and kidney tissues isolated from LPS+IFNγ‐injected mice with or without CA77.1 pretreatment. Scale bar = 50 µm. (E,F) Plasma ALT, AST, Cr, and BUN detection (n = 3) in PBS, LPS+IFNγ, or CA77.1‐treated LPS+IFNγ‐injected mice. (G) Kaplan‐Meier survival analysis between mice injected with LPS+IFNγ with or without CA77.1 pretreatment (n = 7). (H) Heatmap depicting the relative levels of IL‐1β, IL‐6, and TNF‐α in the serum of *WT* and *LAMP2a^−/−^* mice injected with PBS or LPS+IFNγ. (I) Representative images of lung, liver, and kidney tissues isolated from PBS‐ or LPS+IFNγ‐treated *WT* and *LAMP2a^−/−^* mice. Scale bar = 50 µm. (J,K) Liver ALT (n = 4), AST (n = 4), and serum Cr (n = 4), BUN (n = 5) detection in PBS‐ or LPS+IFNγ‐injected *WT* or *LAMP2a^−/−^* mice. (L) Kaplan‐Meier survival analysis between *WT* or *LAMP2a^−/−^* mice injected with LPS+IFNγ (n = 5). (M) Representative images of lung, liver, and kidney tissues isolated from LPS+IFNγ‐injected mice with or without oxamate pretreatment. Scale bar = 50 µm. (N) Representative images of lung, liver, and kidney tissues isolated from LPS+IFNγ‐treated *LDHA^flox/flox^* and *LDHA^flox/flox^ Lyz2‐Cre* mice. Scale bar = 50 µm. (O) Kaplan–Meier survival analysis between *LDHA^flox/flox^* and *LDHA^flox/flox^ Lyz2‐Cre* injected with LPS+IFNγ (n = 7). Data are shown as mean ± SD. Analysis was performed using one‐way ANOVA (A, C, E‐F and J‐K). *n.s*. stands for not significant.

To validate the role of lactylation in this pathway, we employed oxamate and *LDHA^flox/flox^ Lyz2‐Cre* mice. Oxamate pretreatment in mice significantly alleviated lung, liver, and kidney injury (Figure 8M). Furthermore, *LDHA^flox/flox^ Lyz2‐Cre* mice challenged with IFNγ/LPS exhibited markedly attenuated multi‐organ injury, as evidenced by histopathology, and showed improved survival (Figure 8N,O). In summary, in vivo validation confirms that enhancing CMA activity or blocking lactate production attenuates the inflammatory storm, reduces macrophage death, alleviates multi‐organ injury, and improves survival in septic mice, consistent with our in vitro findings.

## 3 Discussion

Our study identifies the “Kla‐CMA‐CUX1/Pyrin‐PANoptosis” signaling axis, which mechanistically links dysregulated metabolism, post‐translational modification, defective CMA, and inflammatory cell death in septic macrophages. This discovery that CMA orchestrates Pyrin inflammasome‐dependent PANoptosis through CUX1 suppression positions CMA as a decisive molecular switch that calibrates inflammatory responses, rather than merely a homeostatic degradative pathway. The negative regulation of CUX1 by CMA represents a sophisticated mechanism translating septic insults into programmed inflammatory cell fate. These findings not only elucidate a pathogenic cascade driving cytokine storm, but also suggest the therapeutic potential of blocking lactylation or enhancing CMA activity to improve prognosis in septic patients with cytokine storm.

The metabolite lactate, a hallmark of septic metabolic reprogramming, has emerged as a key mediator of immune cell function via lactylation [40, 41]. Beyond its role in histone modifications, lactylation regulates non‐histone proteins such as pyruvate kinase M2 (PKM2) [42] and fatty acid synthase (FASN) [43]. Here, we demonstrate that lactate accumulation in septic macrophages induces lactylation of HSC70, the central chaperone in CMA, at the K561 residue, thereby impairing its function. Genetic blockade of the lactylation site (K561R) restored CMA activity, establishing a causal relationship between lactate‐induced lactylation and CMA suppression. These findings extend the regulatory scope of non‐histone lactylation to the maintenance of proteostasis and identify lactate as an upstream trigger of macrophage dysfunction in sepsis.

CMA, a selective lysosomal degradation pathway, has been increasingly recognized for its role in modulating immune responses and cellular homeostasis under stress conditions. CMA dysfunction is typically associated with chronic age‐related diseases, such as neurodegenerative diseases and metabolic syndromes [44, 45, 46, 47]. Growing evidence highlights the role of CMA in regulating diverse immune and stromal cells. CMA activity is known to govern hematopoietic stem cell maintenance [48], microglial quiescence [25], T cell responsiveness [49], and mesenchymal stromal cell differentiation [26]. CMA dysfunction promotes macrophage inflammation [23, 37], while a sustained inflammatory microenvironment can further suppress CMA activity [25, 26], establishing a vicious cycle that exacerbates disease progression. In the context of septic inflammatory storm, our findings demonstrate deficient CMA activity under inflammatory storm conditions, consistent with previous studies, and establish a causal link between CMA failure and sepsis‐related organ damage and poor prognosis. Conversely, we observed a more severe inflammatory storm in *LAMP2a^−/−^* macrophages, indicating that disrupted CMA intrinsically induces macrophage inflammation. This proposed vicious cycle of “CMA dysfunction” and “macrophage inflammation” can be interrupted by both CMA activators and lactylation inhibitors, thereby improving septic progression. CMA serves as a precision regulator of cellular proteostasis by selectively degrading specific proteins, rather than acting as a non‐specific scavenger. Our study reinforces this concept by revealing that in sepsis, CMA impairment is not part of a generalized autophagy dysfunction, but results from specific inhibition via HSC70 lactylation, leading to the pathological accumulation of its substrate CUX1.

CUX1 is a multifunctional transcription factor involved in diverse cellular processes, including cell cycle and differentiation. In this study, we identify CUX1 as a physiological substrate of CMA, suggesting that CMA helps maintain macrophage homeostasis by continuously degrading CUX1 and keeping its activity at an appropriate level. This regulatory paradigm is consistent with reports that CMA also targets other transcription factors, such as HIF‐1α [50] and NF‐κB [25], underscoring its broader role in modulating transcriptional networks. Under septic conditions, CMA inhibition via the lactate‐HSC70 axis leads to CUX1 accumulation, nuclear translocation, and subsequent transcriptional upregulation of Pyrin. Although elevated NLRP3 expression was also observed during the cytokine storm, we focused on the sensor Pyrin. This decision was based on the established understanding that NLRP3 is a known substrate of CMA [23, 24], and its accumulation could be attributed to compromised CMA activity. In contrast, the regulatory mechanism linking CMA dysfunction to Pyrin upregulation remained unexplored, representing a novel aspect of this signaling axis. Our data support a model in which CUX1‐driven Pyrin expression facilitates Pyrin‐PANoptosome assembly, ultimately triggering PANoptosis in macrophages.

The CMA‐CUX1‐Pyrin pathway likely functions as a double‐edged sword in sepsis. On one hand, macrophage inflammatory cell death driven by this axis serves as a potent antimicrobial defense. By undergoing lytic death, infected macrophages directly eliminate intracellular pathogen niches, releasing damage‐associated molecular patterns (DAMPs) and pathogen‐associated molecular patterns (PAMPs) that amplify local inflammation and recruit additional immune effectors [51, 52, 53]. On the other hand, excessive or systemic activation of this death pathway can become a major driver of the cytokine storm and multi‐organ dysfunction characteristic of severe sepsis. Impairment of CMA‐mediated CUX1 suppression, whether due to disease progression or genetic predisposition, may shift this balance toward pathology. Thus, the CMA‐CUX1 axis represents a finely tuned determinant of immune homeostasis in sepsis, requiring precise calibration to ensure effective defense without collateral damage.

Therapeutically, our study suggests multiple intervention points. First, pharmacological modulators of CMA could be used to fine‐tune macrophage PANoptosis. Early administration of CMA agonists might temper hyperinflammation by enhancing CUX1 degradation, whereas transient CMA inhibition could, in specific contexts, bolster antimicrobial PANoptosis. Alternatively, directly targeting the HSC70‐CUX1 interaction or blocking CUX1‐driven Pyrin expression may allow more precise control of this pathway with reduced systemic effects. Importantly, the efficacy of any such intervention will likely depend on the septic etiology and timing of administration, underscoring the need for context‐specific therapeutic design.

Several limitations of this study should be acknowledged. First, the human cohort was small (n = 5 per group), and no independent replication cohort was available for the human findings. The study was therefore underpowered to draw definitive conclusions about the clinical utility of the Kla‐CMA‐CUX1‐Pyrin axis. Second, in this study, the LPS+IFNγ model, representing sterile and exogenous inflammatory stimulation rather than authentic polymicrobial infection, was used for mechanistic dissection of the signaling axis, whereas the CLP model was used to confirm that the core axis is engaged in authentic polymicrobial sepsis. Conclusions derived from each model should be interpreted within its respective context, and the two models are complementary rather than interchangeable. Third, only male mice were used in this study. Given that sex is a well‐recognized biological variable influencing immune responses and sepsis outcomes, our findings may not be fully generalizable to female mice. Future studies incorporating both sexes and sex‐stratified analyses will be needed to determine whether the HSC70 lactylation‐CMA‐CUX1‐Pyrin axis operates similarly in females. Fourth, direct biochemical evidence for HSC70 K561 lactylation is incomplete, as the writer enzyme responsible for this modification has not been identified and an in vitro lactylation assay could not be reliably reconstituted. Lastly, CA77.1 is not a selective CMA activator, and the sufficiency of CUX1 was not tested by gain‐of‐function or epistasis experiments because lentiviral expression of the large CUX1 protein in primary BMDMs was technically unfeasible. These limitations should be considered when interpreting the generalizability of our findings.

Beyond the limitations discussed above, several key questions remain for future research. First, the specific septic signals that regulate the CMA‐CUX1 interaction, such as metabolic alterations, oxidative stress, or bacterial toxins, remain to be defined. Second, it is essential to determine whether CUX1 directly binds the promoter of Pyrin and other PANoptosome components, and to map its full transcriptional network in activated macrophages, for example by employing ChIP‐seq. Finally, exploring the role of this axis in other cell types involved in sepsis, such as neutrophils and endothelial cells, and exploring its safety and efficacy in patients rather than in preclinical models will clarify its broader contribution to sepsis pathophysiology.

In summary, we describe a signaling axis that links metabolic stress to the clinical progression of sepsis. Specifically, both lactate‐induced K561 lactylation of HSC70 and inflammatory storm itself disrupt CMA, leading to CUX1 accumulation, increased Pyrin expression, and subsequent PANoptosis in macrophages. This reveals a critical link between selective autophagy and inflammatory cell death. This pathway offers new mechanistic insights and identifies potential therapeutic targets for septic shock and related inflammatory disorders.

## 4 Materials and Methods

### 4.1 Patient Samples

Peripheral blood mononuclear cells (PBMCs) and plasma were extracted from whole blood of healthy (n = 5) and sepsis patients (n = 5) from Shanghai General Hospital between July 2025 and December 2025 (see Table 1). The sample collection was pre‐approved by the Ethics Committee of Shanghai General Hospital (2025KS520). Written informed consent was also obtained. Sepsis was defined in accordance with the Third International Consensus Definitions for Sepsis and Septic Shock (Sepsis‐3) [54].

**TABLE 1:** Clinical characteristics of healthy and sepsis patients.

Parameters	Healthy (n = 5)	Sepsis (n = 5)
Age (years)	73.20 ± 9.04	67.60 ± 8.41
Gender (male/female)	4/1	4/1
APACHE II	N/A	25.40 ± 4.10
qSOFA	N/A	10.60 ± 1.67
GCS	N/A	13.80 ± 1.79

*Notes*: Data are shown as mean ± standard deviation.

Abbreviations: APACHE II, Acute Physiology and Chronic Health Evaluation; GCS, Glasgow Coma Scale; N/A, not applicable; qSOFA, Quick Sequential Organ Failure Assessment.

### 4.2 Experimental Animals

Male C57BL/6 wild‐type mice (aged 8–10 weeks) were purchased from Shanghai Legen Biotechnology Co., Ltd. *LAMP2a^−/−^* mice and KFERQ‐Dendra transgenic mice were donated by Professor Shuxian Dong (Clinical Transformation Research Institute of Shanghai General Hospital). *AIM2^−/−^*, *NLRP3^−/−^*, *Pyrin^−/−^*, *ZBP1^−/−^*, *LDHA^flox/flox^* and *LDHA^flox/flox^ Lyz2‐Cre* mice were all introduced from Cyagen Biotechnology Co., LTD. All animals were raised in a 12 h light/dark cycle with free access to food and water in a temperature‐controlled facility. All animal experiments performed in this study were in accordance with the Guides for the Care and Use of Laboratory Animals (National Academy of Sciences, China), and all procedures were approved by Shanghai General Hospital Clinical Center Laboratory Animal Welfare & Ethics Committee (Ethical approval number: 2025AWS552).

For treatment with the CMA activator, mice were intraperitoneally single‐injected with CA77.1 (MedChemexpress, #HY‐134923, 15 mg/kg) or vehicle once a day for 5 consecutive days. For LPS or/and IFNγ treatment, all animals were randomly divided into four groups: (i) phosphate‐buffered saline (PBS) group, (ii) IFNγ (20 µg, intraperitoneal injection; Thermo Fisher Scientific, #315‐05) group, (iii) LPS (20 mg/kg, intraperitoneal injection; Sigma, #L2630) group, and (iv) LPS + IFNγ group. Twenty‐four hours after the injection, blood, lung, liver, and kidney were harvested for later analysis, after sevoflurane anesthesia or/and euthanasia.

For cecal ligation and puncture (CLP) procedures [55], C57BL/6 mice were anesthetized with inhaled sevoflurane (3% mixed with oxygen) and subjected to midline laparotomy, followed by cecum ligation 1 cm distal to the ileocecal valve and transmural puncture with a 22G needle and feces extrusion, after which the abdomen was closed. Sham‐operated mice underwent identical procedures excluding ligation and perforation steps. Saline (50 µL/g) and buprenorphine (0.05 µg/g) were then subcutaneously given to all surgical mice.

### 4.3 Cell Culture and Drug Treatment

Primary mouse bone marrow‐derived macrophages (BMDMs) were collected from femurs and tibias of mice of the indicated genotype. After centrifugation and erythrolysis, BMDMs were resuspended in free culture medium. Then, the obtained cells were cultured in RPMI 1640 medium with 10% fetal bovine serum (FBS, Gibco), 10 ng/mL M‐CSF (Thermo Fisher Scientific, #315‐02), and 1× penicillin‐streptomycin (New Cell & Molecular Biotech Co., Ltd, #C100C5) for 7 days before subsequent experiments. Human embryonic kidney 293T (HEK293T) cell lines were purchased from the Cell Bank of the Chinese Academy of Sciences and cultured in DMEM containing 10% FBS and 1× penicillin‐streptomycin. Cells were all cultured in a CO2 incubator at 37°C.

Cells were treated with the following concentrations of drug according to the group arrangement where indicated unless otherwise noted: 20 µM of CA77.1 (Selleck Technology, #S6395), 10 nM of sodium oxamate (Selleck Technology, #S6871), 10 mM of 3‐Methyladenine (3‐MA, MedChemexpress, #HY‐19312), 50 µM of chloroquine (CQ, MedChemexpress, #HY‐17589A), 10 µM of MG‐132 (MedChemexpress, #HY‐13259C), 10 mM of ammonium chloride (NH_4_Cl, MedChemexpress, #HY‐Y1269C), 500 ng/mL of LPS (Sigma, #L2630), 50 ng/mL of IFNγ (Thermo Fisher Scientific, #315‐05), 100 µg/mL of cycloheximide (MedChemexpress, #HY‐12320), 5 µM of LDC7559 (Selleck, #S9622), 5 µM of Z‐VAD‐FMK (Selleck, #S7023), 20 µM of Necrostatin‐1(MedChemexpress, #HY‐15760). CA77.1 and sodium oxamate were administered to cells before (12 h for CA77.1 and 2 h for sodium oxamate) LPS or/and IFNγ treatment, while 3‐MA, chloroquine, MG‐132, and ammonium chloride were given to cells 4–6 h before harvest. Cycloheximide was added to cells at the indicated time. LDC7559, Z‐VAD‐FMK, and Necrostatin‐1 were administered to cells 1 h before IFNγ/LPS treatment.

### 4.4 SYTOX Staining Assay

BMDMs were cultured and treated with the indicated drug, while PBMCs were extracted from whole blood of human or mouse using lymphocyte separation medium (Dakewe Biotech Co., Ltd. #7111011, #7211011), followed by incubation with SYTOX Green for 10 min (Thermo Fisher Scientific, #S7020). Finally, cells were observed with an inverted DMI8 microscope system (Leica, Germany) or applied to C6 Plus for flow cytometry.

### 4.5 Western Blot

The cells were harvested and lysed in RIPA lysis buffer (New Cell & Molecular Biotech Co., Ltd., #WB3100) and sample loading buffer. Next, the cell lysates were separated by SDS‐polyacrylamide gel electrophoresis (PAGE) and transferred onto polyvinylidene difluoride (PVDF) membranes (Merck, #IPVH00010). Nonspecific binding was blocked by Blocking Buffer (New Cell & Molecular Biotech Co., Ltd, #P30500), then membranes were incubated with primary antibodies overnight against: LAMP2a (Thermo Fisher Scientific, #51‐2200, 1:1000), LAMP2a (Abcam, ab18528, 1:1000), HSC70 (Abcam, ab51052, 1:1000), HSC70 (Santa cruz, sc‐7298, 1:1000), HSC70 (Proteintech, #10654‐1‐AP, 1:1000), LAMP1 (Abcam, ab25245,1:1000), LAMP1 (CST, #15665, 1:1000), LC3B (Merck, #L7543, 1:1000), p62 (CST, #5114S, 1:1000), ATG3 (CST, #3415T, 1:1000), ATG12 (CST, #4180T, 1:1000), ATG5 (Abmart, #T55766, 1:1000), ATG7 (Abmart, #T57051, 1:1000), Beclin (Novus Biologicals, #NB500‐249, 1:1000), caspase‐3 (CST, #14220S, 1:1000), caspase‐3 (Proteintech, #19677‐1‐AP, 1:1000), caspase‐7 (CST, #12827S, 1:1000), cleaved caspase‐7 (CST, #8438S, 1:1000), pMLKL (Abcam, ab196436, 1:1000), pMLKL (CST, #37333S, 1:1000), tMLKL (CST, 37705S, 1:1000), tMLKL (Proteintech, #21066‐1‐AP, 1:1000), tMLKL (Proteintech, #66675‐1‐Ig, 1:1000), pRIPK1 (Affinity, #AF2398, 1:1000), pRIPK1 (Proteintech, #66854‐1‐Ig, 1:1000), tRIPK1 (CST, #3493S, 1:1000), caspase‐1 (CST, #24232S, 1:1000), cleaved caspase‐1 (CST, #89332S, 1:1000), GSDMD (CST, #39754S, 1:1000), GSDMD (Abcam, ab209845, 1:1000), cleaved GSDMD (CST, #34667S, 1:1000), AIM2 (CST, #53491S, 1:1000), NLRP3 (Proteintech, #68102‐1‐Ig, 1:1000), Pyrin (Abcam, ab195975, 1:1000), Pyrin (Abcam, ab214772, 1:1000), Pyrin (Proteintech, #24280‐1‐AP, 1:1000), ZBP1 (Adipogen, #AG‐20B‐0010‐C100, 1:1000), RIPK3 (Abmart, #TA7942, 1:1000), CASP8 (CST, #4790, 1:1000), CUX1 (Abcam, ab307821, 1:1000), CUX1 (Proteintech, #68449‐1‐Ig, 1:1000), Kla (PTM BIO, PTM‐1401,1:1000), LDHA (Proteintech, #19987‐1‐AP, 1:1000), LDHB (HUABIO, #HA724336, 1:1000), β‐tubulin (Absin, #abs830032, 1:2000), Flag (CWBIO, CW0287M, 1:2000), His (CWBIO, CW0286M, 1:2000). The membranes were washed and then incubated with appropriate horseradish peroxidase (HRP)—conjugated secondary antibodies (Abmart, Anti‐Rabbit/Mouse/Rat IgG‐HRP, M21002S/M21001S/M212162) for 2 h. Proteins were eventually visualized using NcmECL Ultra enhanced chemiluminescent detection kit (New Cell & Molecular Biotech Co., Ltd, #P10300).

### 4.6 Quantitative Real‐Time Polymerase Chain Reaction

Total RNA was extracted from samples using TRIzol (Thermo Fisher Scientific, #15596018CN) and reverse‐transcribed into cDNA with HiScript II RT SuperMix (Vazyme, #R223‐01). Real‐time quantitative polymerase chain reaction (PCR) was performed using the Universal SYBR qPCR Mix (EnzyArtisan, #Q204‐01). Primers were listed in Table 2.

**TABLE 2:** Primers used for the real‐time RT‐PCR analysis.

Primers	Sequence
18S‐mouse‐F	5’‐ GTAACCCGTTGAACCCCATT ‐3’
18S‐mouse‐R	5’‐ CCATCCAATCGGTAGTAGCG ‐3’
LAMP2a‐mouse‐F	5’‐ ACAGTATTCTACAGCTCAAGACTGC ‐3’
LAMP2a‐mouse‐R	5’‐ CACTAGAATAAGTACTCCTCCCAGAG ‐3’
HSPA8‐mouse‐F	5’‐ CCGATGAAGCTGTTGCCTATGG ‐3’
HSPA8‐mouse‐R	5’‐ CCAAGGGAAAGAGGAGTGACATC ‐3’
LAMP1‐mouse‐F	5’‐ CCAGGCTTTCAAGGTGGACAGT ‐3’
LAMP1‐mouse‐R	5’‐ GGTAGGCAATGAGGACGATGAG ‐3’
AIM2‐mouse‐F	5’‐ TAACTTCATGGAGGTCACCAGTTC ‐3’
AIM2‐mouse‐R	5’‐ GGAGTTTCCCTGGCTCTCTTAAC ‐3’
NLRP3‐mouse‐F	5’‐ ATTACCCGCCCGAGAAAGG ‐3’
NLRP3‐mouse‐R	5’‐ TCGCAGCAAAGATCCACACAG ‐3’
Pyrin‐mouse‐F	5’‐ CGACCCAGGAGTCTTGAAATTA ‐3’
Pyrin‐mouse‐R	5’‐ GTCCTTCCATTCAGAGTAGCTGTT ‐3’
ASC‐mouse‐F	5’‐ CTGCTCAGAGTACAGCCAGAAC ‐3’
ASC‐mouse‐R	5’‐ CTGTCCTTCAGTCAGCACACTG ‐3’
RIPK1‐mouse‐F	5’‐ GACTGTGTACCCTTACCTCCGA ‐3’
RIPK1‐mouse‐R	5’‐ CACTGCGATCATTCTCGTCCTG ‐3’
IL‐1β‐mouse‐F	5’‐ TGGACCTTCCAGGATGAGGACA ‐3’
IL‐1β‐mouse‐R	5’‐ GTTCATCTCGGAGCCTGTAGTG ‐3’
IL‐6‐mouse‐F	5’‐ TACCACTTCACAAGTCGGAGGC ‐3’
IL‐6‐mouse‐R	5’‐ CTGCAAGTGCATCATCGTTGTTC ‐3’
TNF‐α‐mouse‐F	5’‐ GGGTTGTACCTTGTCTACTCCCAG ‐3’
TNF‐α‐mouse‐R	5’‐ GAGATAGCAAATCGGCTGACG ‐3’
CUX1‐mouse‐F	5’‐ GAGAATACGAGCAGACCCTGAAG ‐3’
CUX1‐mouse‐R	5’‐ TCTGTGTCTCTTGCAGCTTTCTCT ‐3’
Mouse‐Pyrin‐1‐CHIP‐F	5’‐ TATAGACAGTGGATATACATCAATGCA ‐3’
Mouse‐Pyrin‐1‐CHIP‐R	5’‐ CCCCATTGTCCCAGAAGTGTC ‐3’
Mouse‐Pyrin‐2‐CHIP‐F	5’‐ CAGTCTGGATGAATTGGTGAGC ‐3’
Mouse‐Pyrin‐2‐CHIP‐R	5’‐ GTGTGCTTTATAGAGGCCAGAGAC ‐3’
Mouse‐Pyrin‐3‐CHIP‐F	5’‐ CAAGAGCTTGAGGCAGAAATG ‐3’
Mouse‐Pyrin‐3‐CHIP‐R	5’‐ AAAGTGAGTTTCAGAACAGCAAAAG ‐3’
Mouse‐Pyrin‐4‐CHIP‐F	5’‐ TGACAATCAATGTCAGCCTTCAC ‐3’
Mouse‐Pyrin‐4‐CHIP‐R	5’‐ CCCTGGCTTCCTAGGTCCAC ‐3’
Mouse‐Pyrin‐5‐CHIP‐F	5’‐ CTGGAAGGTCTGGCTGTCAATC ‐3’
Mouse‐Pyrin‐5‐CHIP‐R	5’‐ GATTCAGAGTCTTTTCCAGTGGG ‐3’

### 4.7 Biochemical Analysis

The levels of IL‐1β (#88‐7013), IL‐6 (#88‐7064), and TNF‐α (#88‐7324), were measured using ELISA kits purchased from Thermo Fisher Scientific. Serum and extracellular lactate levels were detected using a lactate assay kit (Nanjing Jiancheng Bioengineering Institute, #A019‐2‐2), while extracellular lactate dehydrogenase (LDH) levels were measured by the LDH assay kit (#A020‐2‐2). Serum creatinine (#C011‐2‐1), blood urea nitrogen (BUN, #C013‐2‐1), alanine aminotransferase (ALT) (#C009‐2‐1), and aspartate aminotransferase (AST) (C010‐2‐1) were all analyzed using respective assay kits purchased from Nanjing Jiancheng Bioengineering Institute according to the manufacturer's instructions.

### 4.8 RNA Interference

*CUX1* siRNA was purchased from Shanghai QEgene Biotechnology Co., Ltd. with the following sequence: si‐NC sense:5’‐UUCUCCGAACGUGUCACGUTT‐3’; si‐*CUX1*‐1^#^: 5’‐CCGCAACAGUAUUGGCAAATT‐3’; si‐*CUX1*‐2^#^: 5’‐GGGAAACACUAGAAGAGUATT‐3’. *HSC70* siRNA was purchased from Genomeditech with the following sequence: si‐*HSC70*‐1^#^ sense:5’‐ GAGGGAAUUGACUUCUAUA‐3’; si‐*HSC70*‐2^#^: 5’‐ CCUCCAUGGUUCUGACAAA‐3’; si‐*HSC70*‐3^#^: 5’‐ GAGUUCAAGCGAAAGCACA‐3’. *ATG5* siRNA was purchased from Genomeditech with the following sequence: si‐*ATG5*‐1^#^ sense:5’‐ GGUGAUGAUUCACGGGAUA‐3’; si‐*ATG5*‐2^#^: 5’‐ AUUUCGUUGAUCACUUGAC‐3’; si‐*ATG5*‐3^#^: 5’‐ CAUGGAAUAUCCUCCAGAA‐3’. *ATG7* siRNA was purchased from Genomeditech with the following sequence: si‐*ATG7*‐1^#^ sense:5’‐ GAUUCACCUUCCUAGCGAA‐3’; si‐*ATG7*‐2^#^: 5’‐ GAGGGUUCAACAUGAGCAU‐3’; si‐*ATG7*‐3^#^: 5’‐ CAAGGGCUAUUACUACAAU‐3’. *LDHB* siRNA was purchased from Genomeditech with the following sequence: si‐*LDHB*‐1^#^ sense:5’‐ CAGUGGAUAUUCUGACUUA‐3’; si‐*LDHB*‐2^#^: 5’‐ GUGUUGGAAGACAAGCUCA‐3’; si‐*LDHB*‐3^#^: 5’‐ GAUAAAGAUUACUCUGUGA‐3’. Lipofectamine 2000 reagent (Thermo Fisher Scientific, #11668019) was used to transfect siRNA into BMDMs. After transfection for 6 h, the medium of BMDMs was replaced with fresh medium. One day later, they were co‐stimulated with IFNγ/LPS for 24 h and then harvested for further assessment of CUX1 and Pyrin expression.

### 4.9 Plasmid Transfection

Both the HSC70 plasmids (HSC70‐wildtype and the other seven mutants of HSC70) and the CUX1 plasmids (CUX1‐wildtype and one mutant of CUX1) were constructed by Wuhan Miaoling Biotechnology Co., Ltd. 293T cells were seeded in 24‐well plates or 10 cm dishes and then transfected with wild‐type or mutant plasmids using EZ Trans (Shanghai Li Ji Biotechnology Co., LTD, #AC04L091) for 36 h. The cells were collected for further Western Blot, immunoprecipitation, or immunofluorescence assays.

### 4.10 Co‐Immunoprecipitation (Co‐IP) Assay

First, BMDMs were gently washed with ice‐cold PBS, after which they were lysed with IP lysis buffer (Thermo Fisher Scientific, #87787) containing Protease and Phosphatase Inhibitor Cocktail (New Cell & Molecular Biotech Co., Ltd, #P002) and then scraped into a centrifuge tube. After 30 min of lysis on ice, the tubes were centrifuged to remove debris and collect the supernatant. For each group, we took 10% of the supernatant as Input, and the remaining was determined as the IP group. Then, the protein A/G agarose beads (Thermo Fisher Scientific, #20422) or EZview Red ANTI‐FLAG M2 Affinity Gel (Merck, #F2426) were washed with wash buffer before being added to the lysate. For the endogenous Co‐IP assay, the lysate was incubated with capture antibodies (specific antibody against the target protein) or IgG (CST, #5946) and protein A/G beads, while for exogenous Co‐IP, the lysate was incubated with ANTI‐FLAG Gel only. After the overnight incubation at 4°C with gentle rotation, the supernatant was discarded. The beads were washed with wash buffer 3–4 times afterward, and finally eluted to get the target protein and interacting partners for further analysis.

### 4.11 Immunofluorescence Staining

BMDMs were seeded into a 24‐well plate pre‐coated with coverslips sterilized by UV ray beforehand. After respective drug treatment, BMDMs were fixed with 4% paraformaldehyde (PFA) for 10 min, followed by 3 washes with PBS. Then 0.1% Triton X‐100 was applied to permeabilize cells. After being washed with PBS for 3 times, BMDMs were incubated with blocking buffer (3% bovine serum albumin in PBS) for 60 min. Next, BMDMs were incubated with primary antibodies at 4°C overnight, followed by incubation with Alexa Fluor secondary antibodies for 2 h at room temperature. Subsequently, the coverslips seeded with BMDMs were removed from a 24‐well plate and placed onto slides, with BMDMs stained and sealed with DAPI Fluoromount‐G (Yeasen, 36308ES20). Lastly, the cells were observed with a confocal microscope system (TCS SP8; Leica). The following primary antibodies were used: anti‐LAMP2a (Abcam, ab18528), anti‐HSC70 (Santa cruz, sc‐7298), anti‐LAMP1 (Abcam, ab25245), anti‐CUX1 (Abcam, ab307821), anti‐Pyrin (Abcam, ab195975), anti‐ASC (Abcam, ab175449), anti‐cleaved caspase‐3 (Santa cruz, #373730), anti‐pMLKL (CST, #37333S). The following secondary antibodies were used: Alexa Fluor 488 goat anti‐rabbit IgG (H+L) (Beyotime Biotechnology, #A0423), Alexa Fluor 647 goat anti‐mouse IgG (H+L) (Beyotime Biotechnology, #A0473), Alexa Fluor 555 goat anti‐mouse IgG (H+L) (Beyotime Biotechnology, #A0460), Cy3 goat anti‐rat IgG (H+L) (Beyotime Biotechnology, #A0507), Alexa Fluor 647 donkey anti‐goat IgG (H+L) (Abcam, ab150131).

### 4.12 Seahorse Assay

BMDMs were plated into the XFe96 plate at a density of 40 000 cells per well. 24 h before oxygen consumption rate (OCR) and extracellular acidification rate (ECAR) measurement, BMDMs were stimulated with LPS or/and IFNγ. Then, the medium was replaced with XF DMEM base medium with the addition of pyruvate (1 mM), glutamine (2 mM), and glucose (10 mM), after which cells were put into a CO_2_‐free incubator at 37°C for 1 h. Both OCR and ECAR were measured using a 96‐well Seahorse XFe96 Bioanalyzer (Agilent Technologies). For OCR measurement, once in the reader, the plate was sequentially injected with oligomycin (1.5 µM), FCCP (1 µM), and rotenone and antimycin A (0.5 µM) from the XF Cell Mito Stress Test Kit (Agilent, #103015‐100). ECAR was measured after sequential injection of rotenone (0.5 µM) and 2‐deoxyglucose (50 mM) from the XF Glycolytic Rate Assay Kit (Agilent, #103344‐100).

### 4.13 Chromatin Immunoprecipitation (ChIP)

After 10 min of 1% formaldehyde‐induced cross‐linking of DNA with protein, the reaction was quenched with glycine. Then, BMDMs were lysed and sonicated to shear DNA into 200–1000 bp fragments. The following ChIP steps were performed using the EZ‐ChIP Kit (Merck, #17‐371) according to the protocol, with an anti‐CUX1 antibody (Abcam, ab307821) or normal IgG as a negative control. After reverse crosslinking and DNA purification, the precipitated DNA was analyzed by quantitative real‐time PCR (qPCR) using primers targeting the Pyrin promoter (Table 2). The primer pair Mouse‐Pyrin‐5, which targets a distal region outside the core promoter, was used as a negative control region. Enrichment was calculated relative to the IgG control and normalized to input.

### 4.14 RNA Sequencing and Analysis

Total RNA extracted from BMDMs was subjected to RNA sequencing (Illumina HisSeq Sequencing System) performed by Shanghai Xuran Biotechnology Co., Ltd (Shanghai, China). Data were presented as the expected number of Reads Per Kilobase of transcript per million base pairs sequenced (RPKM). Gene expression levels were calculated as the expected number of Fragments Per Kilobase of transcript sequence per million base pairs sequenced (FPKM) by DESeq2 (version 1.16.1). Differentially expressed genes (DEGs, *p*‐value ≤ 0.05 and |log2FoldChange|≥1) were included for functional analysis using GO, KEGG, DisGeNET, and Reactome pathway databases.

### 4.15 Molecular Docking and Molecular Dynamics Simulations

Template‐free protein–protein docking was performed using HDOCK to generate candidate HSC70‐CUX1 complexes. The top‐ranked extended CUT3 model (CUX1 1102–1209) had an HDOCK score of ‐236.24 and retained a core position close to the original CUT3 pose, and it was therefore selected as the candidate complex. The repaired HSC70 (1‐646)/CUX1 (1102‐1209) complex served as the common starting structure for both the unmodified and K561‐lactylated systems. Separate docking scores were not calculated for the two states.

Molecular dynamics (MD) simulations were performed using OpenMM 8.6 with CUDA mixed precision, the Amber14 force field, and the TIP3P water model, with 0.15 M NaCl. Long‐range electrostatics were treated with PME, and a 1.0‐nm cutoff was used for nonbonded interactions. The system was maintained at 300 K and 1 bar. Lactylation parameters were obtained from https://doi.org/10.1038/s41589‐025‐02107‐8. Neither nucleotide nor Mg^2^^+^ was included, and CUX1 was modeled as a fragment. Both systems were equilibrated for 1 ns followed by 100 ns production simulations, with frames saved every 10 ps (10 000 frames per state). One trajectory was generated per state. Conformational analyses used all production frames, with NBD defined as residues 1–386, SBDβ as 395–508, and the lid as 509–607. RMSF was calculated after Kabsch alignment on the NBD or lid. Contacts were defined by a heavy‐atom distance cutoff of 4.5 Å, with 4.0‐ and 5.0‐Å cutoffs used to verify the direction of changes. Temporal consistency was described using five consecutive 20 ns blocks.

### 4.16 Statistical Analysis

All values were shown as mean ± SD. GraphPad Prism 8.0 software was used for statistical analysis and graphing. Normality of data distribution was assessed using the Shapiro–Wilk test before applying parametric tests. Statistical significance was determined by unpaired two‐tailed Student's *t*‐tests for two groups or one‐way ANOVA followed by Tukey's post hoc test for three or more groups. Kaplan–Meier analysis was used to compare differences in survival rates between groups. Pearson correlation was used to analyze the correlation between two groups. *p*< 0.05 was considered statistically significant.

## Author Contributions

Conceptualization: Ying Chen, Yan Zhang, Pei Wang, and Jiamin Ma; methodology: Ying Chen, Yan Zhang, Jiamin Ma, Xinru Zhao, and Yi Li; investigation: Jiamin Ma, Pei Wang, Xinru Zhao, Yi Li, Zhenghao Deng, and Xueqing Meng; formal analysis: Ying Chen, Yan Zhang, Pei Wang, and Jiamin Ma; writing – original draft: Ying Chen, Yan Zhang, and Jiamin Ma; writing – review and editing: Jiali Zhu and Liangfang Yao; funding acquisition: Jinbao Li and Liangfang Yao; supervision: Jiali Zhu, Jinbao Li, and Liangfang Yao.

## Ethics Approvals

Patient samples collection is pre‐approved by the Ethics Committee of Shanghai General Hospital (2025KS520). Written informed consent is also obtained. All animal experiments performed in this study are in accordance with the Guides for The Care and Use of Laboratory Animals (National Academy of Sciences, China) and all procedures are approved by Shanghai General Hospital Clinical Center Laboratory Animal Welfare & Ethics Committee (2025AWS552).

## Conflicts of Interest

The authors declare no conflicts of interest.

## Supporting information




**Supporting File 1**: advs78586‐sup‐0001‐SuppMat.pdf.

**Supporting File 2**: advs78586‐sup‐0002‐SuppMat.pptx.

**Supporting File 3**: advs78586‐sup‐0003‐SuppMat.xlsx.

## Data Availability

All data supporting the findings of this study are available within the article and its Supplementary Information. The RNA‐seq data have been deposited in the NCBI SRA under BioProject accession PRJNA1531364. The IP‐MS and lactylproteomics data have been deposited in the ProteomeXchange Consortium via the iProX partner repository under accession number PXD084510. All other data are available from the corresponding author upon reasonable request.
