# Supplementary material for: Lactylation of HSC70 Impairs Chaperone‐Mediated Autophagy to Promote Pyrin Inflammasome‐Dependent PANoptosis in Macrophages During Sepsis
Source: Adv Sci (Weinh). 2026 Nov 3:e78586. Online ahead of print. doi: 10.1002/advs.78586 (PMC13731082; doi:10.1002/advs.78586)
Supplement: Supplementary file 1 [file ADVS-9999-e78586-s001.pdf]

Supplementary figures and figure legends

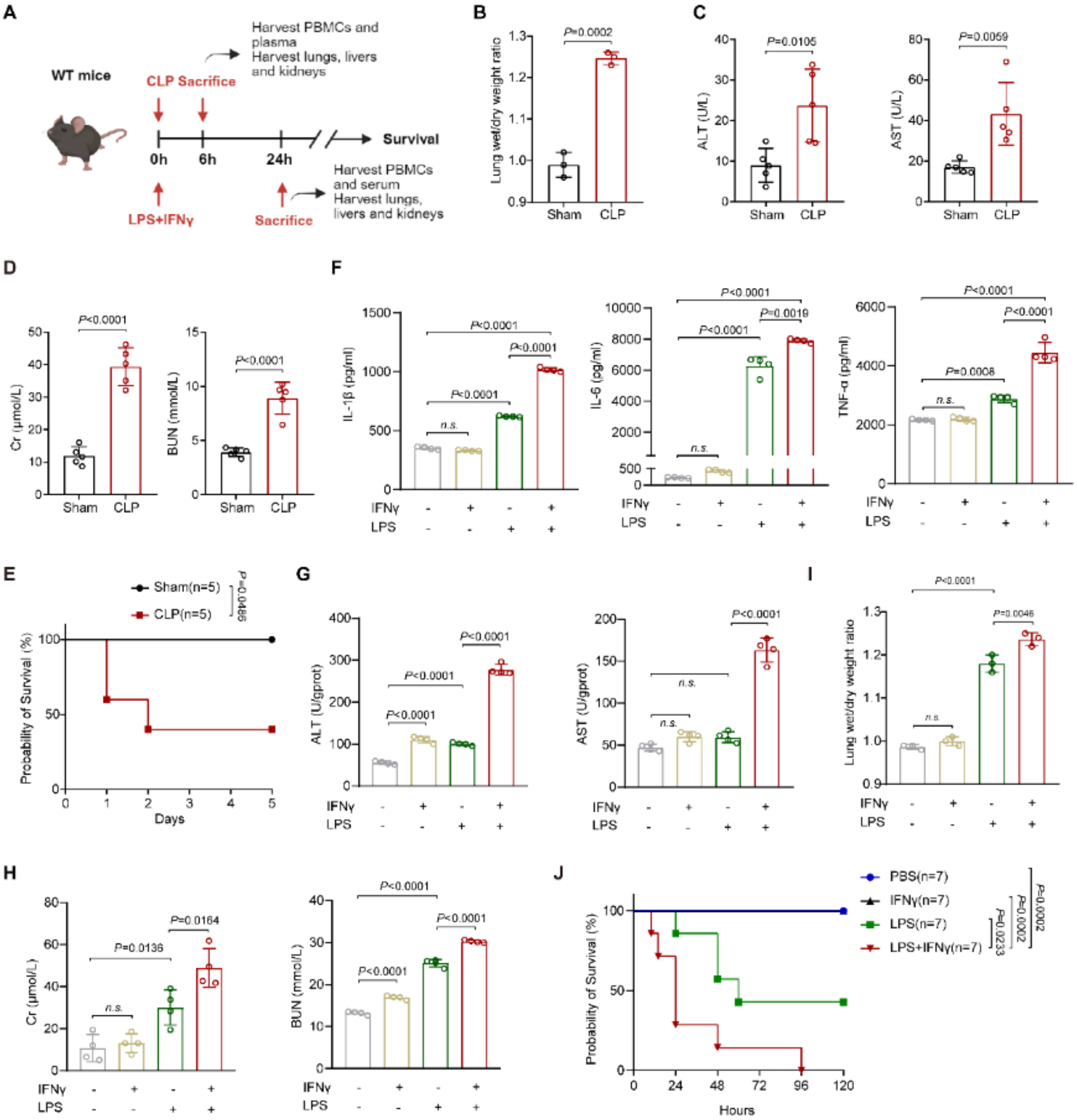

Figure S1 Inflammatory storm aggravates multiple organ dysfunction and compromises survival in sepsis

(A) Schematic diagram showing the treatment to cecal ligation and puncture (CLP) and lipopolysaccharide + interferon γ (LPS+IFNγ) intraperitoneally injected mice

(B) Lung wet/dry ratio tested after CLP modelling compared with sham controls (n=3).

(C-D) Plasma ALT, AST, creatinine (Cr) and BUN detection (n=5) after CLP modelling compared with sham controls.

(E) Kaplan-Meier survival analysis between sham and CLP mice (n=5).

(F) Enzyme-linked immunosorbent assay (ELISA) test of murine serum IL-1β, IL-6 and TNF-α 24 h after PBS, IFNγ, LPS or LPS+IFNγ injection (n=4).

(G-H) Liver ALT and AST, and serum Cr, BUN detection (n=4) in PBS, IFNγ, LPS or LPS+IFNγ-injected mice.

(I) Lung wet/dry ratio tested in PBS, IFNγ, LPS or LPS+IFNγ-injected mice (n=3).

(J) Kaplan-Meier survival analysis between PBS, IFNγ, LPS or LPS+IFNγ-injected mice (n=7).

Data are shown as mean ± SD. Analysis was performed using the two-tailed Student's t tests (B-D) or one-way ANOVA (F-I). *n.s.* stands for not significant.

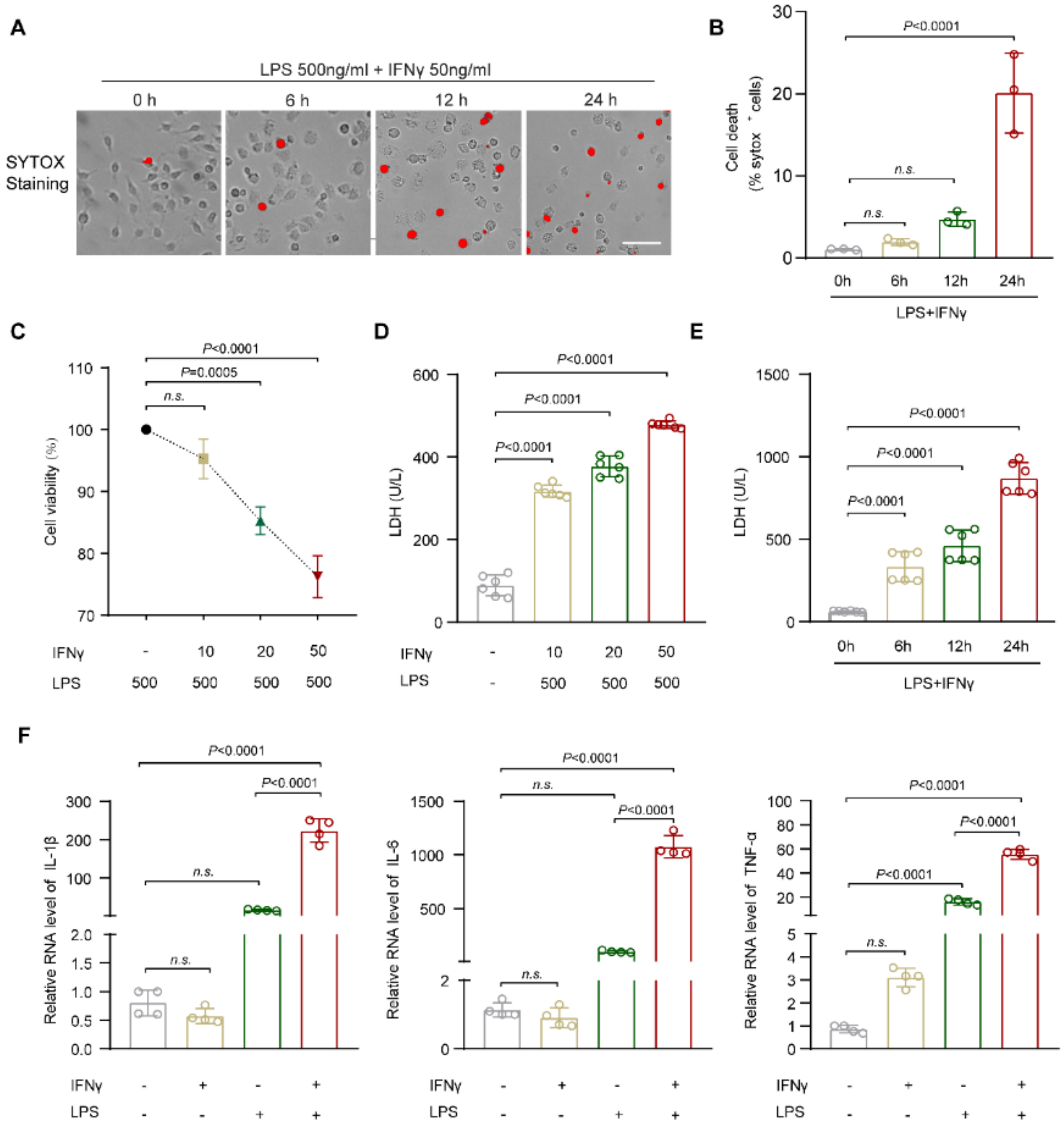

Figure S2 IFNγ/LPS co-stimulation induces cell membrane damage and inflammatory cytokine storm in macrophages

(A-B) Percent of BMDMs that are dead under the stimulation of LPS+IFNγ with indicated time with 500ng/ml of LPS and 50ng/ml of IFNγ using SYTOX staining (n=3). Scale bar=50 μm.

(C) CCK-8 assay for detection of cell viability in BMDMs treated with LPS coupled with IFNγ of different concentration as indicated for 24 h (n=3).

(D-E) LDH release into supernatants detected by LDH kit in BMDMs stimulated by LPS+IFNγ with indicated concentration for 24 h and with indicated time with 500ng/ml of LPS and 50ng/ml

of IFNγ (n=6).

(F) Relative mRNA levels of *IL-1β*, *IL-6* and *TNF-α* in BMDMs treated with PBS, IFNγ, LPS or LPS+IFNγ (n=4).

Data are shown as mean ± SD. Analysis was performed using one-way ANOVA (B-F). *n.s.* stands for not significant.

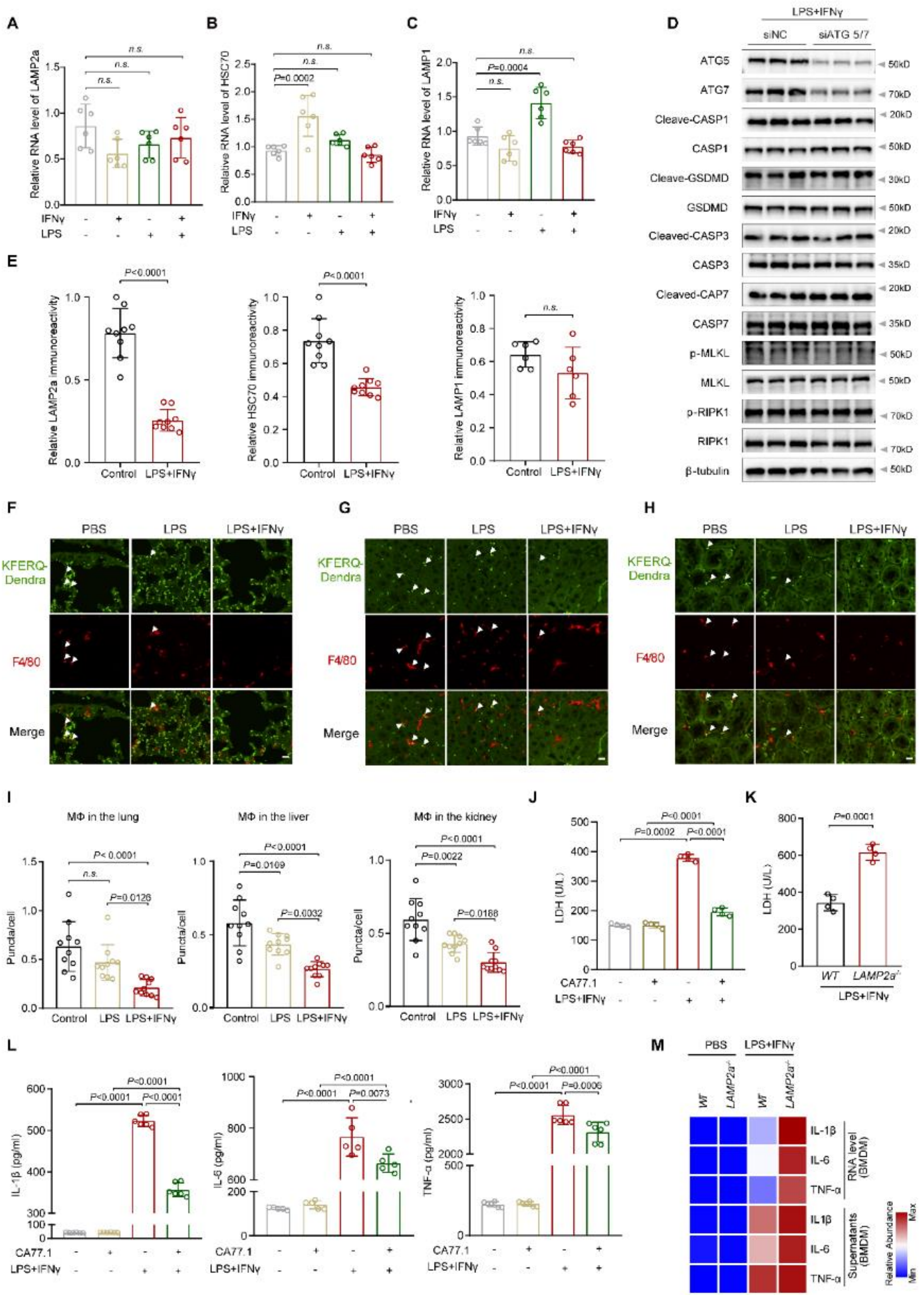

Figure S3 CMA downregulation upon IFNγ/LPS co-stimulation promotes inflammatory storm in macrophages

(A-C) Statistical analyses of mRNA expression of *LAMP2a* (A), *HSC70* (B) and *LAMP1* (C) in

BMDMs treated with PBS, IFNγ, LPS or LPS+IFNγ as indicated (n=6).

(D) Immunoblotting analysis of pro- and cleaved CASP1, pro- and cleaved GSDMD; pro- and cleaved CASP3, pro- and cleaved CASP7; pRIPK1 and tRIPK1, pMLKL and tMLKL, ATG5 and ATG7 in LPS+IFNγ treated siNC or siATG5/7 knockdown BMDMs.

(E) Statistical analyses of relative immunoreactivity of LAMP2a (n=9), HSC70 (n=9) and LAMP1 (n=6) in BMDMs with or without 24 h LPS+IFNγ stimulation. Scale bar=5 μm.

(F-H) Representative images of lung (F), liver (G) and kidney (H) tissues isolated from KFERQ-Dendra2 mice injected with PBS, LPS or LPS+IFNγ as indicated with co-staining with F4/80 for indication of macrophages. Arrowheads indicate the puncta co-localized with macrophages. Scale bar=100 μm.

(I) Statistical analyses of puncta per cell in macrophages in lungs, livers and kidneys isolated from KFERQ-Dendra2 mice (n=10).

(J) LDH release into supernatants in BMDMs stimulated by PBS, CA77.1, LPS+IFNγ or CA77.1-LPS+IFNγ (n=4).

(K) LDH release into supernatants of *WT* and *LAMP2a*$^{-/-}$ BMDMs stimulated by LPS+IFNγ (n=4).

(L) Elisa assay of IL-1β (n=6), IL-6 (n=5) and TNF-α (n=6) in the supernatants of BMDMs treated with PBS, CA77.1, LPS+IFNγ or CA77.1- LPS+IFNγ.

(M) Heatmap depicting the relative levels of IL-1β, IL-6 and TNF-α in the supernatants of *WT* and *LAMP2a*$^{-/-}$ BMDMs stimulated by PBS or LPS+IFNγ and their relative mRNA expression in *WT* and *LAMP2a*$^{-/-}$ BMDMs stimulated by PBS or LPS+IFNγ.

Data are representative of at least three independent experiments. Data are shown as mean ± SD. Analysis was performed using the two-tailed Student's t tests (E and K) or one-way ANOVA (A-C,

I-J and L). *n.s.* stands for not significant.

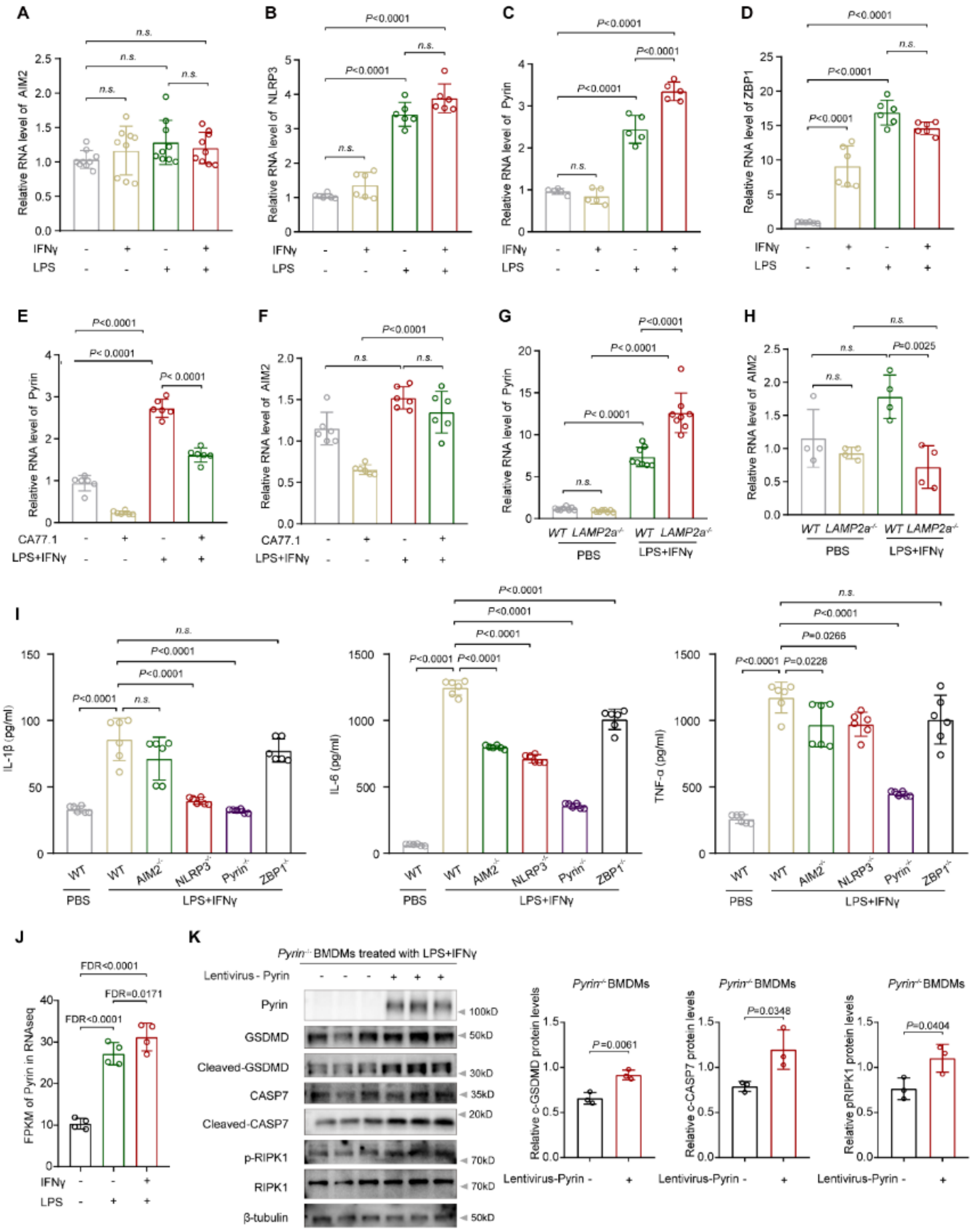

Figure S4 Pyrin is the most involved sensor protein in IFNγ/LPS co-stimulation induced PANoptosis

(A-D) Relative mRNA expression of other sensor proteins as *AIM2* (A, n=9), *NLRP3* (B, n=6), *Pyrin* (C, n=5) and *ZBP1* (D, n=6) in BMDMs treated with PBS, IFNγ, LPS or LPS+IFNγ.

(E-F) Relative mRNA expression of *Pyrin* (E, n=6) and *AIM2* (F, n=6) in BMDMs treated with PBS, CA77.1, LPS+IFNγ or CA77.1- LPS+IFNγ

(G-H) Relative mRNA expression of *Pyrin* (G, n=8) and *AIM2* (H, n=4) in in *WT* and *LAMP2a*$^{-/-}$ BMDMs treated by PBS or LPS+IFNγ.

(I) Elisa assay detection of IL-1β, IL-6 and TNF-α in the supernatants of *WT*, *AIM2*$^{-/-}$, *NLRP3*$^{-/-}$, *Pyrin*$^{-/-}$, *ZBP1*$^{-/-}$ BMDMs stimulated with PBS or LPS+IFNγ (n=6).

(J) FPKM values of Pyrin analyzed using DESeq2 with Wald test (n=4). Control vs LPS+IFNγ: log2FC = 2.203, FDR= 6.09E-106. LPS vs LPS+IFNγ: log2FC = 0.334, FDR = 0.017. Control vs LPS: log2FC = 1.870, FDR = 9.18E-60.

(K) Immunoblotting and densitometric analysis of pro- and cleaved GSDMD; pro- and cleaved CASP7; pRIPK1 and tRIPK1 in *Pyrin*$^{-/-}$ BMDMs transfected with or without lentiviral Pyrin (n=3).

Data are representative of at least three independent experiments. Data are shown as mean ± SD. Analysis was performed using the two-tailed Student's t tests (K), one-way ANOVA (A-I) or Wald test with Benjamini-Hochberg correction for multiple testing (J). *n.s.* stands for not significant.

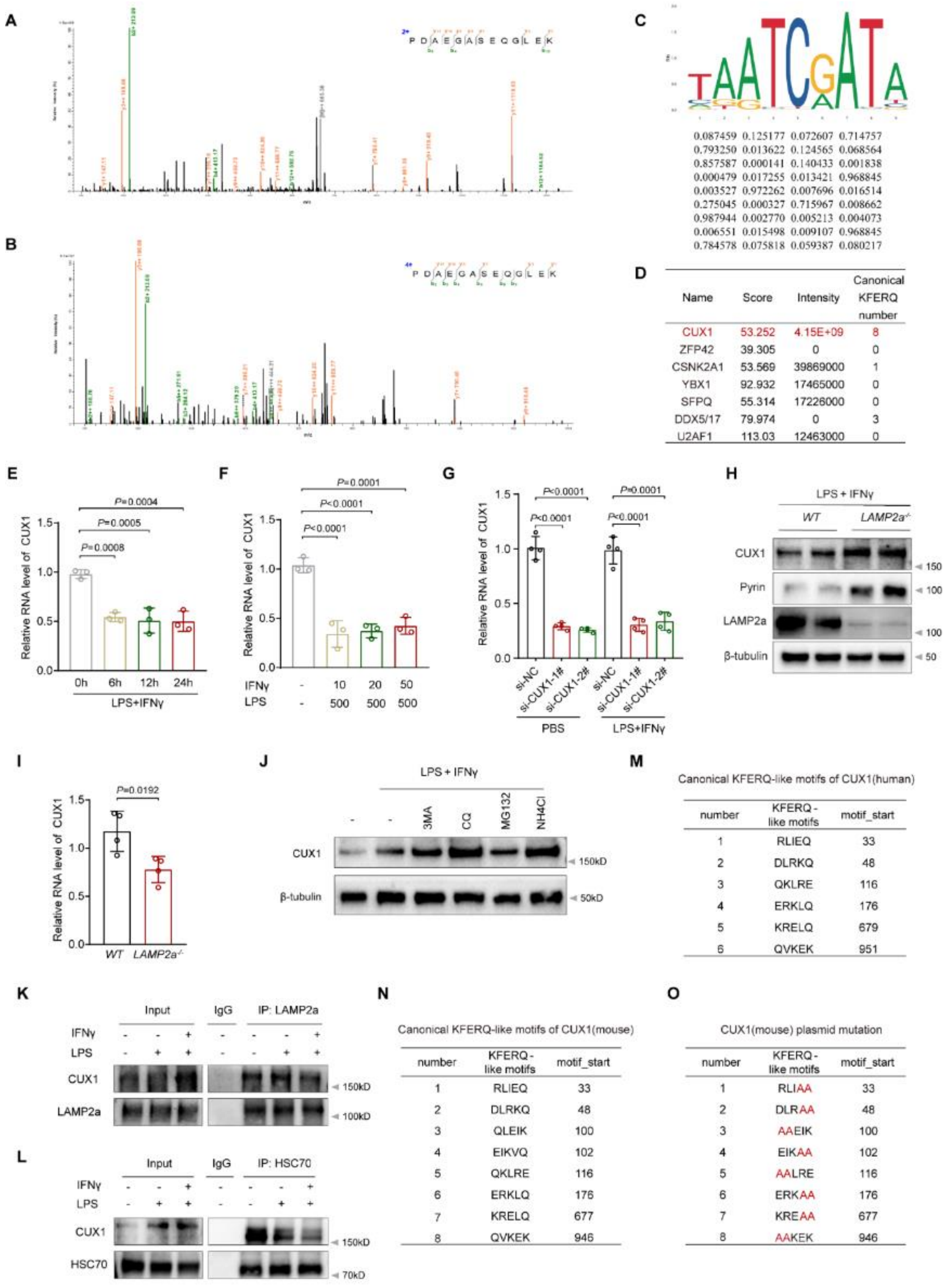

Figure S5 CMA modulates PANoptosis via degradation of the Pyrin-regulating transcription factor CUX1

(A-B) Transcription factor CUX1 found by IP-MS produced by anti-LAMP2a (A) and anti-HSC70

(B) in BMDMs.

(C) The predicted binding site of CUX1 to the promoter of *Pyrin* in JASPAR database.

(D) Summary of the overlapped transcription factor found by IP-MS produced by anti-LAMP2a and anti-HSC70. “Score” and “Intensity” were rendered by IP-MS while “Canonical KFERQ number” was referred to KFERQ-finder V0.8 (https://rshine.einsteinmed.edu/).

(E-F) Relative mRNA expression of *CUX1* in BMDMs treated with LPS+IFNγ with indicated concentration for 24 h and with indicated time with 500ng/ml of LPS and 50ng/ml of IFNγ (n=3).

(G) Relative mRNA expression of *CUX1* in BMDMs transfected with control siRNA or *CUX1* siRNAs stimulated with or without LPS+IFNγ (n=4).

(H) Immunoblotting analysis of CUX1, Pyrin and LAMP2a in *WT* and *LAMP2a*$^{-/-}$ BMDMs treated with LPS+IFNγ.

(I) Relative mRNA expression of *CUX1* in *WT* and *LAMP2a*$^{-/-}$ BMDMs treated with LPS+IFNγ (n=4).

(J) Immunoblotting analysis of CUX1 in BMDMs treated with or without LPS+IFNγ after pretreatment of 3MA, CQ, MG132 and NH4Cl as indicated.

(K-L) Co-immunoprecipitation of LAMP2a with CUX1 (L) and HSC70 with CUX1 (M) from BMDMs treated with PBS, LPS or LPS+IFNγ.

(M-N) List of canonical KFERQ-like motifs of CUX1 in mouse (O) and human (N).

(O) Elaboration of the mutation to the eight KFERQ motifs in mutated mouse CUX1 plasmid.

Data are representative of at least three independent experiments. Data are shown as mean ± SD. Analysis was performed using the two-tailed Student’s t tests (I) or one-way ANOVA (E-G).

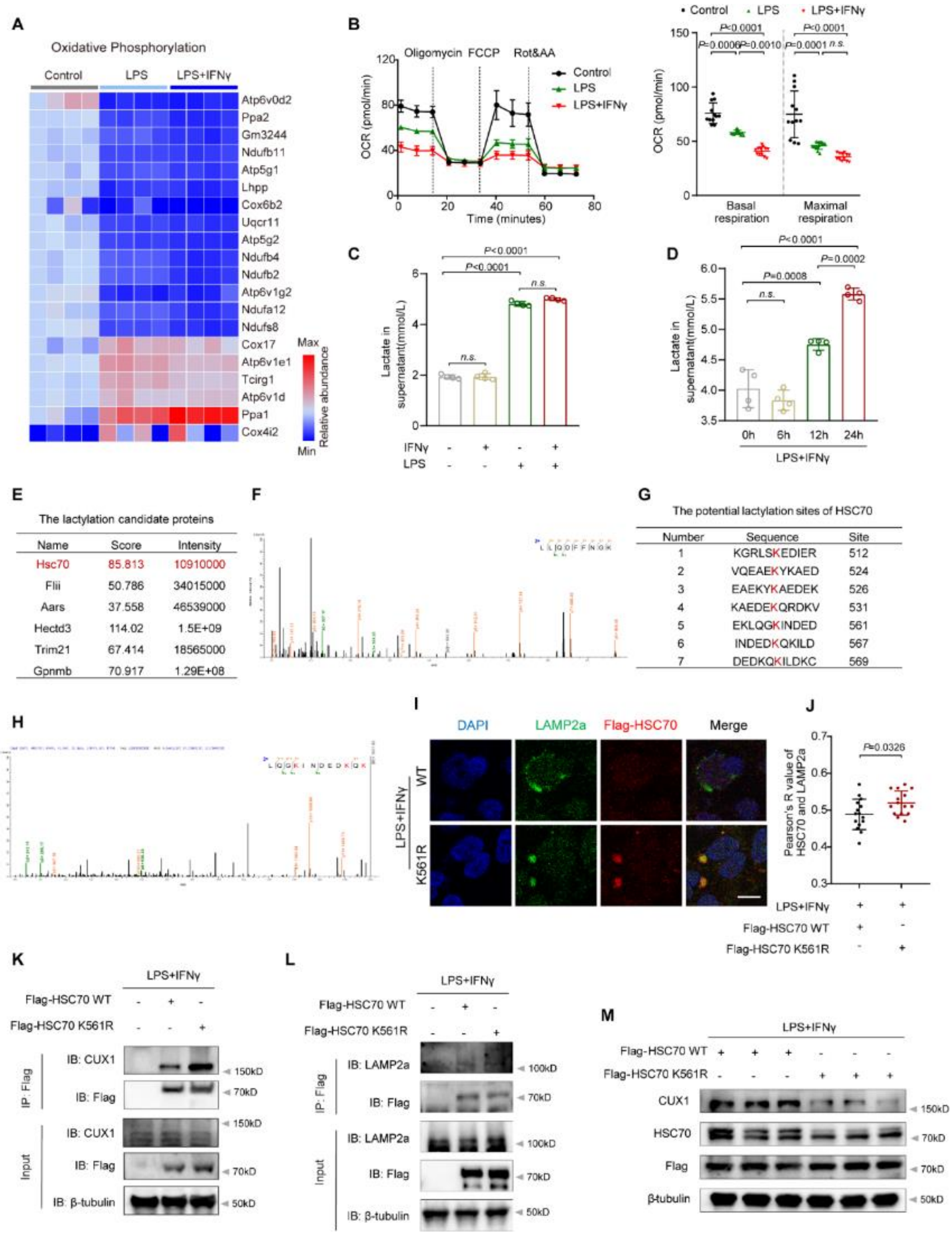

Figure S6 HSC70 is lactylated at K561 upon IFNγ/LPS co-treatment

(A) Heatmap depicting relative mRNA levels of genes involved in the pathway of “Oxidative phosphorylation” in BMDMs treated with PBS, LPS or LPS+IFNγ.

(B) Oxygen consumption rate (OCR, B (left)) assessed by Seahorse Assay in BMDMs treated with

PBS, LPS or LPS+IFNγ, and quantification of basal and maximal respiration (B (right)) (n=12).

(C-D) Lactate levels in supernatants of BMDMs treated with PBS, IFNγ, LPS or LPS+IFNγ (C) and with LPS+IFNγ for indicated time (D) (n=4).

(E) The list of some proteins with high score and intensity (HSC70 included) verified by IP-MS produced by anti-Kla in BMDMs stimulated by LPS+IFNγ.

(F) The protein HSC70 found by IP-MS produced by anti-Kla in BMDMs stimulated by LPS+IFNγ.

(G) The list of seven potential lactylation sites of HSC70 verified by IP-MS produced by anti-HSC70 in BMDMs stimulated with LPS+IFNγ.

(H) The potential lactylation sites K561 of HSC70 verified by IP-MS produced by anti-HSC70 in BMDMs stimulated with LPS+IFNγ.

(I-J) Representative immunofluorescence images of LAMP2a and HSC70 in HEK293T cells with treatment of LPS+IFNγ after transfected with Flag-HSC70 WT or Flag-HSC70 K561R (I), and co-localization analysis of LAMP2a with HSC70 by means of Pearson's R value (J, n=15). Scale bar=10 μm.

(K) Co-immunoprecipitation of Flag/HSC70 with CUX1 from HEK293T cells stimulated with LPS+IFNγ and transfected by Flag-HSC70 WT or Flag-HSC70 K561R.

(L) Co-immunoprecipitation of Flag/HSC70 with LAMP2a from HEK293T cells stimulated with LPS+IFNγ and transfected by Flag-HSC70 WT or Flag-HSC70 K561R.

(M) Immunoblotting analysis of CUX1, HSC70 and Flag in HEK293T cells treated with LPS+IFNγ after transfection of Flag-HSC70 WT or Flag-HSC70 K561R.

Data are representative of at least three independent experiments. Data are shown as mean ± SD.

Analysis was performed using one-way ANOVA (B-D). *n.s.* stands for not significant.

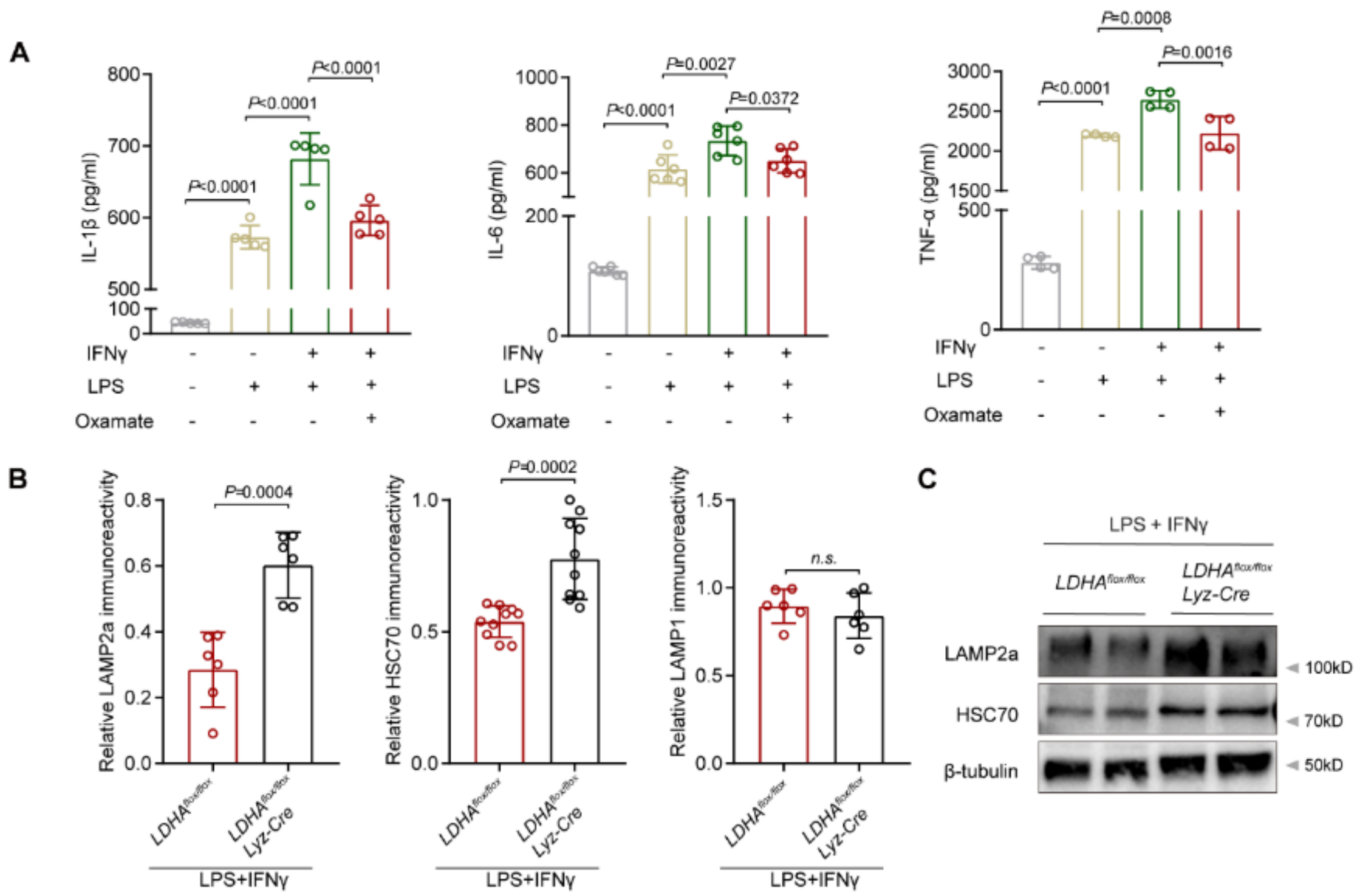

Figure S7 Blockage of lactylation mitigates inflammatory storm and rescues CMA

(A) Elisa assay detection of IL-1β (n=5), IL-6 (n=6) and TNF-α (n=4) in the supernatants of BMDMs treated with PBS, LPS, LPS+IFNγ or oxamate- LPS+IFNγ.

(B) Statistical analyses of relative immunoreactivity of LAMP2a (n=6), HSC70 (n=10) and LAMP1 (n=6) with 24 h LPS+IFNγ stimulation in $LDHA^{flox/flox}$ and $LDHA^{flox/flox}$ *Lyz2-Cre* BMDMs. Scale bar=5 μm.

(C) Immunoblotting analysis of LAMP2a and HSC70 in LPS+IFNγ treated $LDHA^{flox/flox}$ and $LDHA^{flox/flox}$ *Lyz2-Cre* BMDMs.

Data are representative of at least three independent experiments. Data are shown as mean ± SD. Analysis was performed using the two-tailed Student's t tests (B) or one-way ANOVA (A). *n.s.* stands for not significant.

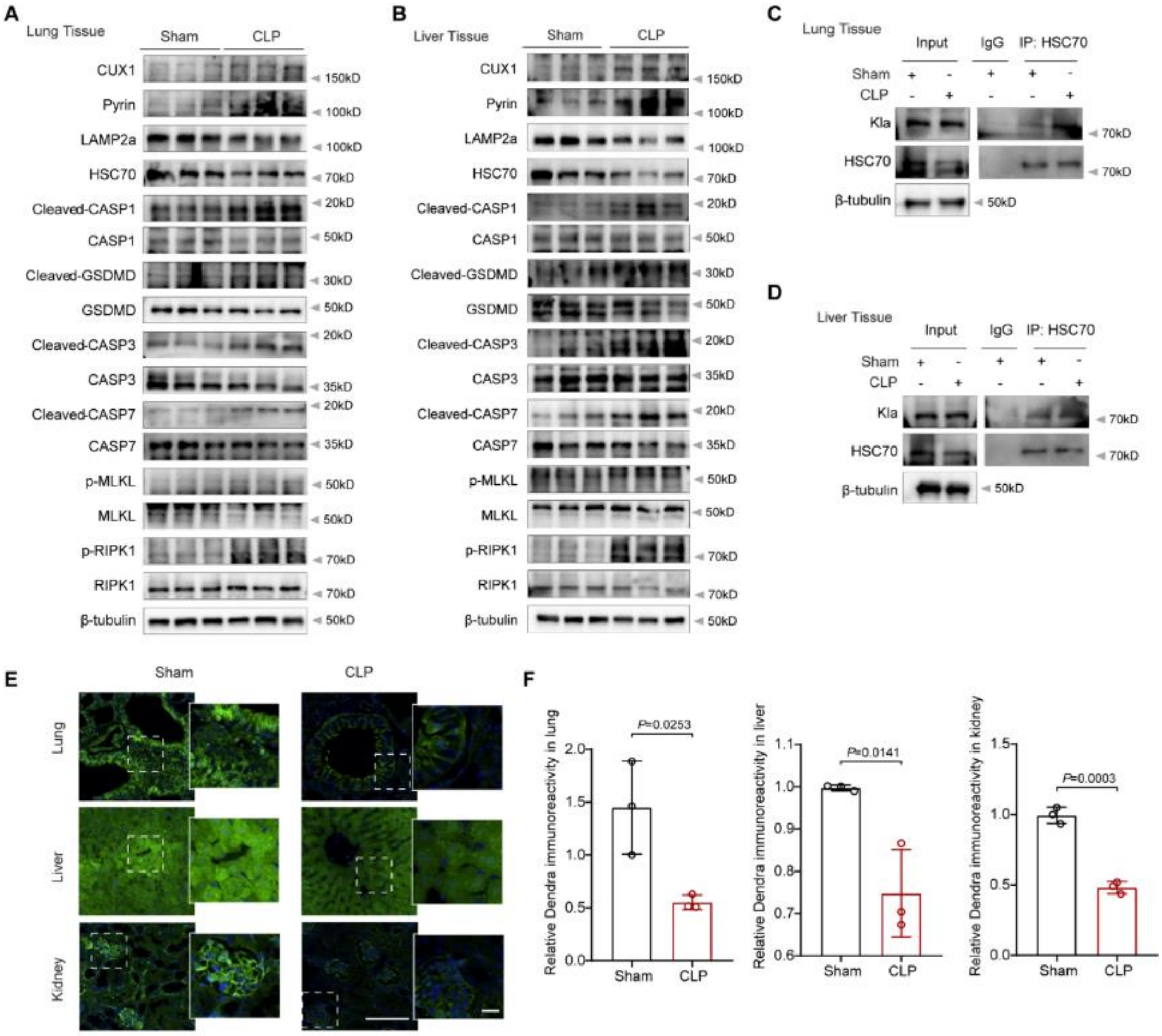

Figure S8 Validation of HSC70 lactylation-CMA-CUX1-Pyrin-PANoptosis axis in CLP model

(A-B) Immunoblotting analysis of pro- and cleaved CASP1, pro- and cleaved GSDMD; pro- and cleaved CASP3, pro- and cleaved CASP7; pRIPK1 and tRIPK1, pMLKL and tMLKL; CUX1, Pyrin, LAMP2a and HSC70 in the lung (A) and liver tissue (B) from sham and CLP mice.

(C-D) Co-immunoprecipitation of HSC70 with Kla from lung tissue (C) and liver tissue (D) of sham and CLP mice.

(E-F) Representative immunofluorescence images of Dendra in the lung, liver and kidney tissue of sham and CLP mice (E), and quantification of relative Dendra immunoreactivity in the lung, liver and kidney tissue (n=3). Scale bar=100 μm.

Data are representative of at least three independent experiments. Data are shown as mean ± SD. Analysis was performed using the two-tailed Student's t tests (F).

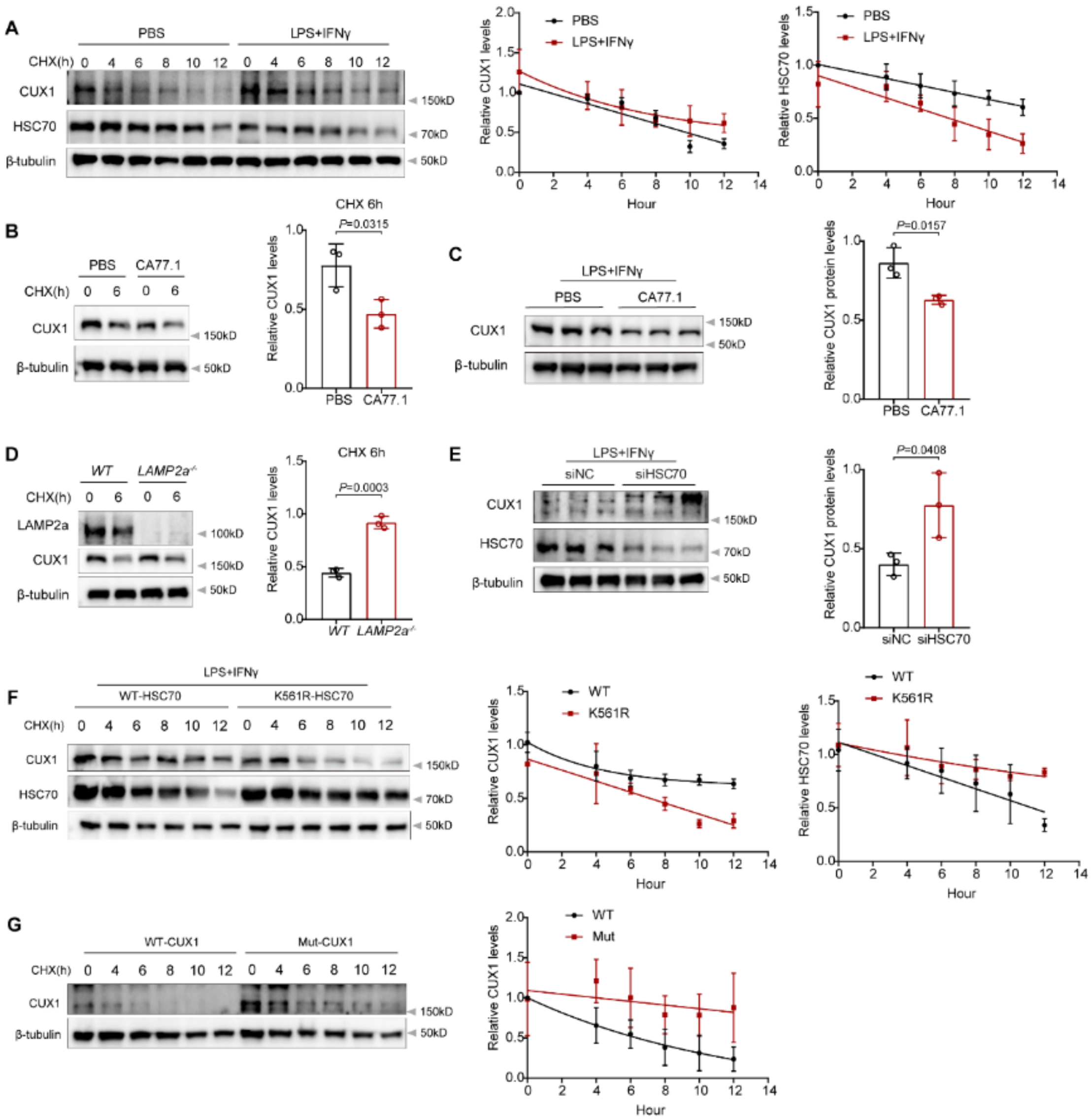

Figure S9 CMA involvement in CUX1 degradation

(A) Immunoblotting and densitometric analysis of CUX1 and HSC70 in PBS or LPS+IFNγ-treated BMDMs with different time of CHX exposure as indicated (n=3).

(B) Immunoblotting and densitometric analysis of CUX1 in PBS or CA77.1-pretreated BMDMs at CHX exposure 0 and 6 hours (n=3).

(C) Immunoblotting and densitometric analysis of CUX1 in PBS or CA77.1-pretreated BMDMs

after stimulated by IFNγ/LPS (n=3).

(D) Immunoblotting and densitometric analysis of CUX1 in *WT* or *LAMP2a*$^{-/-}$ BMDMs at CHX exposure 0 and 6 hours (n=3).

(E) Immunoblotting and densitometric analysis of CUX1 and HSC70 in siNC or siHSC70-treated BMDMs after stimulated by IFNγ/LPS (n=3).

(F) Immunoblotting and densitometric analysis of CUX1 and HSC70 in HEK293T cells after transfected with HSC70-WT or HSC70-K561R with different time of CHX exposure as indicated (n=3).

(F) Immunoblotting and densitometric analysis of CUX1 in HEK293T cells after transfected with CUX1-WT or CUX1-Mut (KFERQ motif mutation) with different time of CHX exposure as indicated (n=3).

Data are representative of at least three independent experiments. Analysis was performed using the two-tailed Student's t tests (B-E).

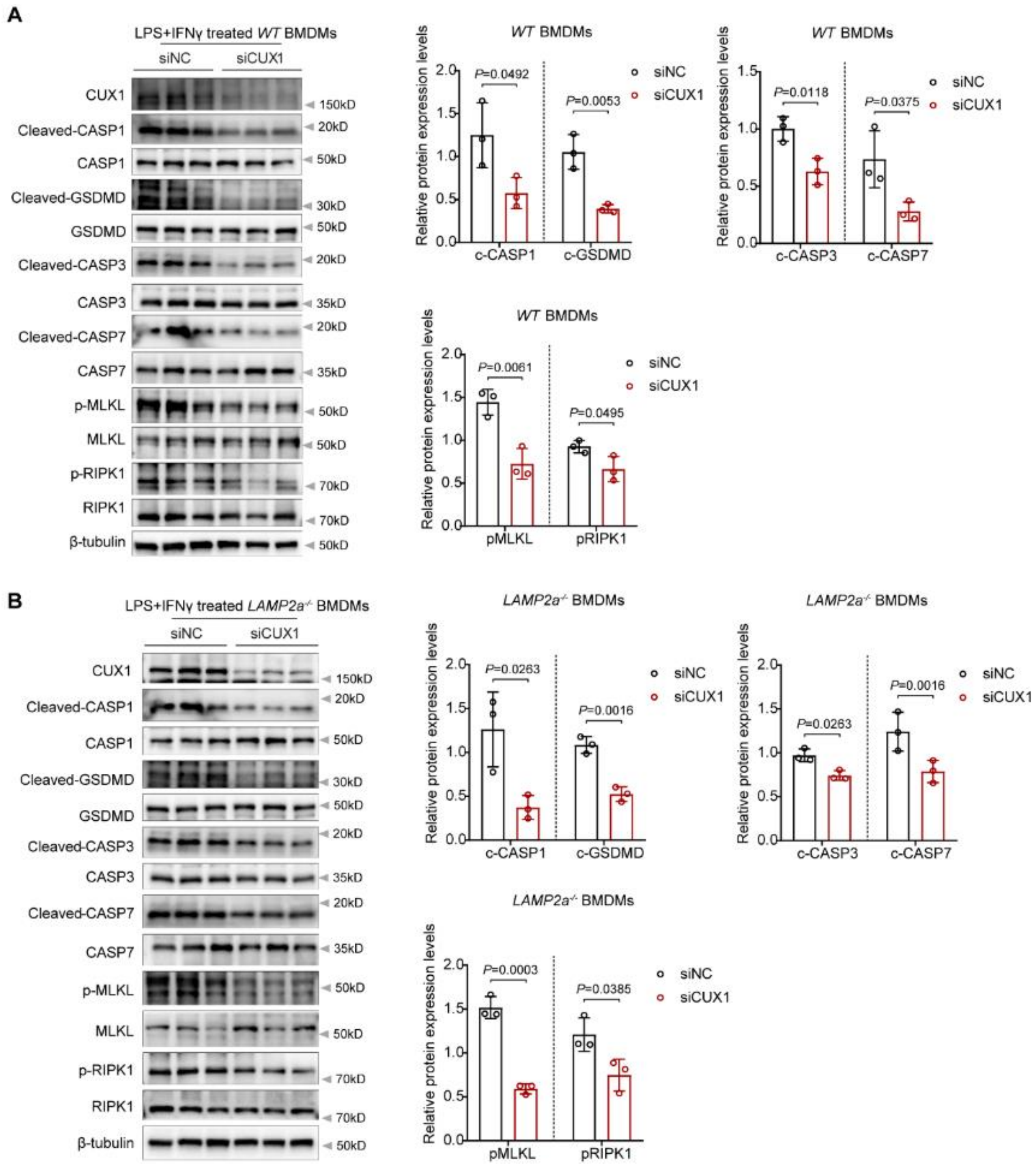

Figure S10 PANoptosis expression after CUX1 knockdown

(A-B) Immunoblotting and densitometric analysis of pro- and cleaved CASP1, pro- and cleaved GSDMD; pro- and cleaved CASP3, pro- and cleaved CASP7; pRIPK1 and tRIPK1, pMLKL and tMLKL; in the *WT* (A) or *LAMP2a*$^{-/-}$ (B) BMDMs with or without CUX1 silencing (n=3).

Data are representative of at least three independent experiments. Analysis was performed using the two-tailed Student's t tests (A-B).

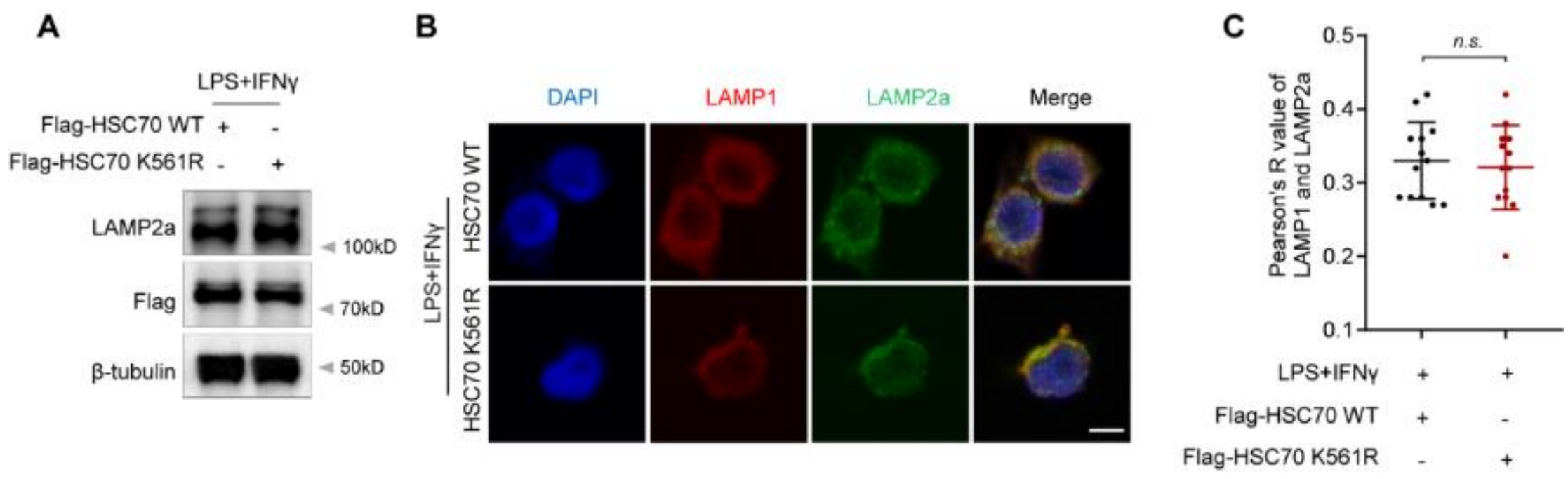

Figure S11 K561R not impact LAMP2a expression and subcellular distribution

(A) Immunoblotting analysis of LAMP2a in the HEK293T cells transfected with WT-HSC70 or K561R-HSC70.

(B-C) Representative immunofluorescence images of LAMP1 and LAMP2a in the HEK293T cells transfected with WT-HSC70 or K561R-HSC70 (B), and co-localization analysis of LAMP2a with LAMP1 by means of Pearson's R value (C, n=13). Scale bar=10 μm.

Data are representative of at least three independent experiments. Data are shown as mean ± SD. Analysis was performed using the two-tailed Student's t tests (C). *n.s.* stands for not significant.

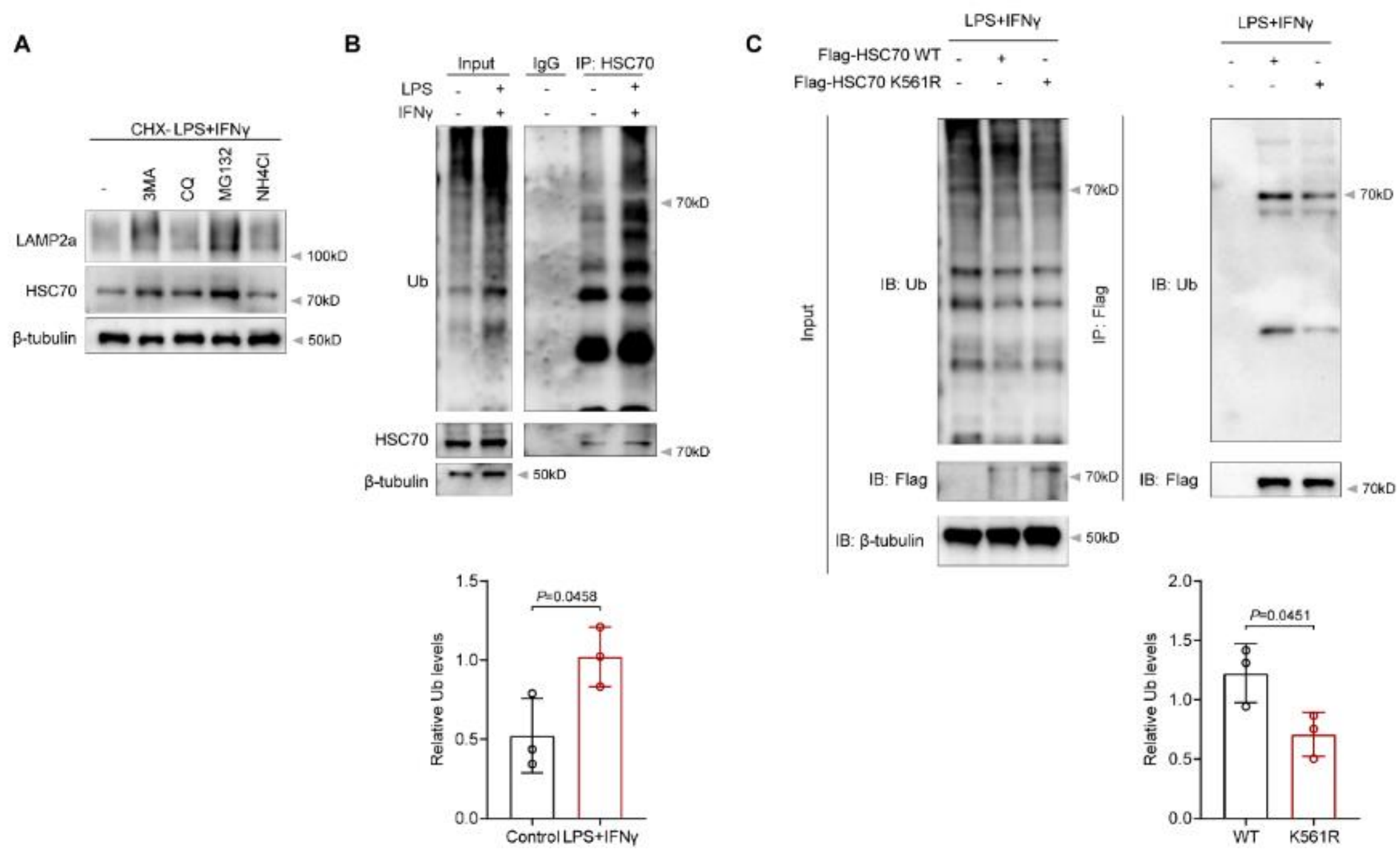

Figure S12 HSC70 Ubiquitination and degradation

(A) Immunoblotting analysis of LMAP2a and HSC70 in BMDMs treated with CHX after

stimulated by LPS+IFNγ after pretreatment of 3MA, CQ, MG132 and NH4Cl as indicated.

(B) Co-immunoprecipitation of HSC70 with Ub in BMDMs treated with or without LPS+IFNγ and densitometric analysis (n=3).

(C) Co-immunoprecipitation of Flag/HSC70 with Ub in HEK293T cells transfected with WT-HSC70 or K561R-HSC70 and densitometric analysis (n=3).

Data are representative of at least three independent experiments. Analysis was performed using the two-tailed Student's t tests (B-C).

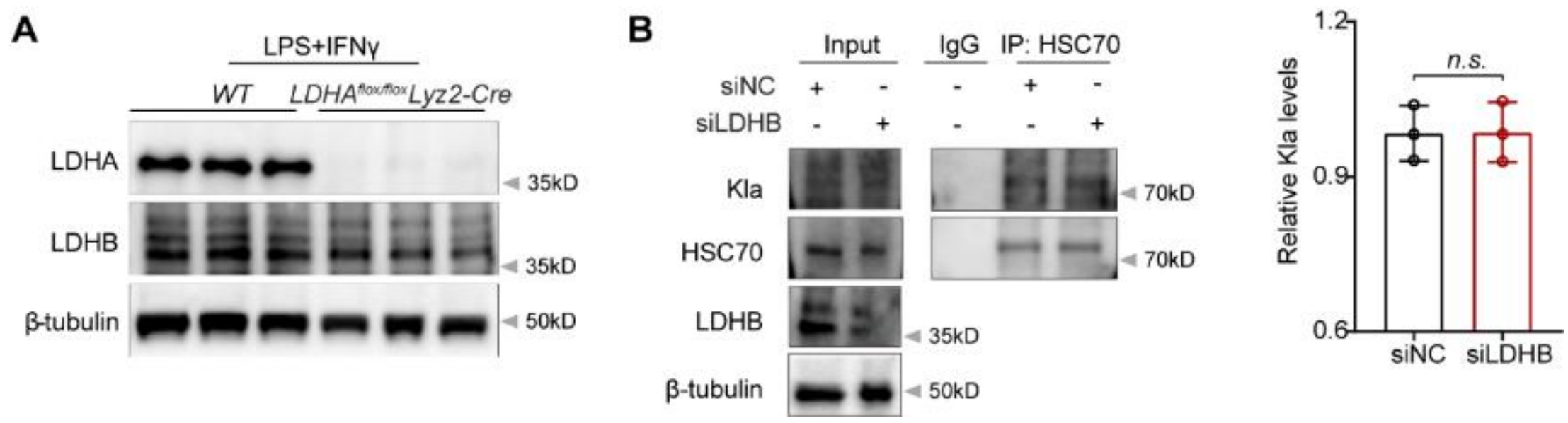

Figure S13 Exclusion of LDHB involvement in lactylation

(A) Immunoblotting analysis of LDHB in *WT* or *LDHA*$^{flox/flox}$ *Lyz2-Cre* BMDMs treated with LPS+IFNγ.

(B) Co-immunoprecipitation of HSC70 with Kla in *LDHA*$^{flox/flox}$ *Lyz2-Cre* BMDMs treated with siNC or siLDHB and densitometric analysis (n=3).

Data are representative of at least three independent experiments. Analysis was performed using the two-tailed Student's t tests (B).

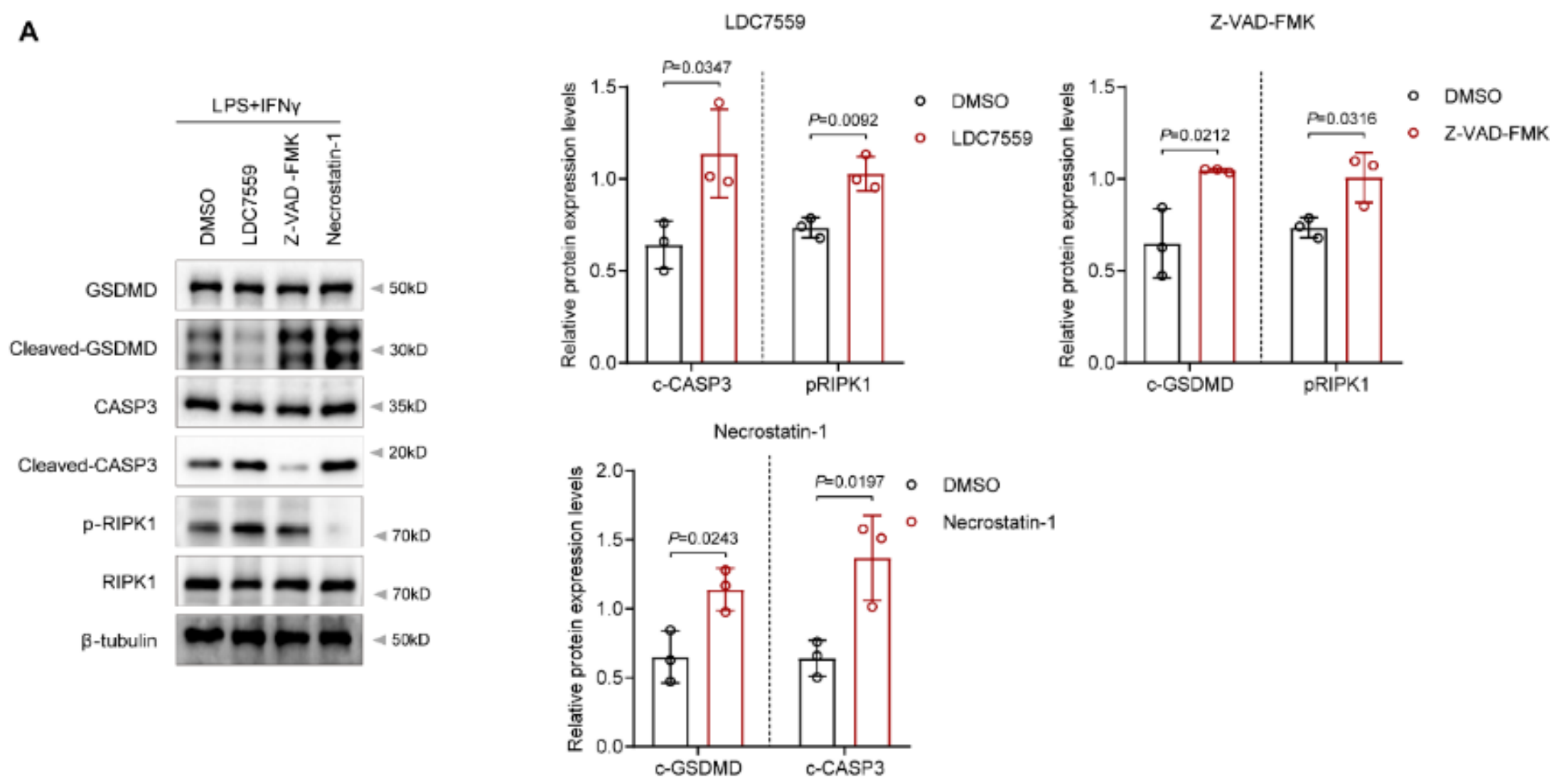

Figure S14 Synergistic PANoptosis in BMDMs

(A) Immunoblotting and densitometric analysis of pro- and cleaved GSDMD, pro- and cleaved CASP3, pRIPK1 and tRIPK1 in the BMDMs treated by LPS+IFNγ with pretreatment of DMSO, LDC7559 (pyroptosis inhibitor), Z-VAD-FMK (pan-apoptosis inhibitor) or Necrostatin-1 (necroptosis inhibitor) (n=3).

Data are representative of at least three independent experiments. Analysis was performed using the two-tailed Student's t tests (A).

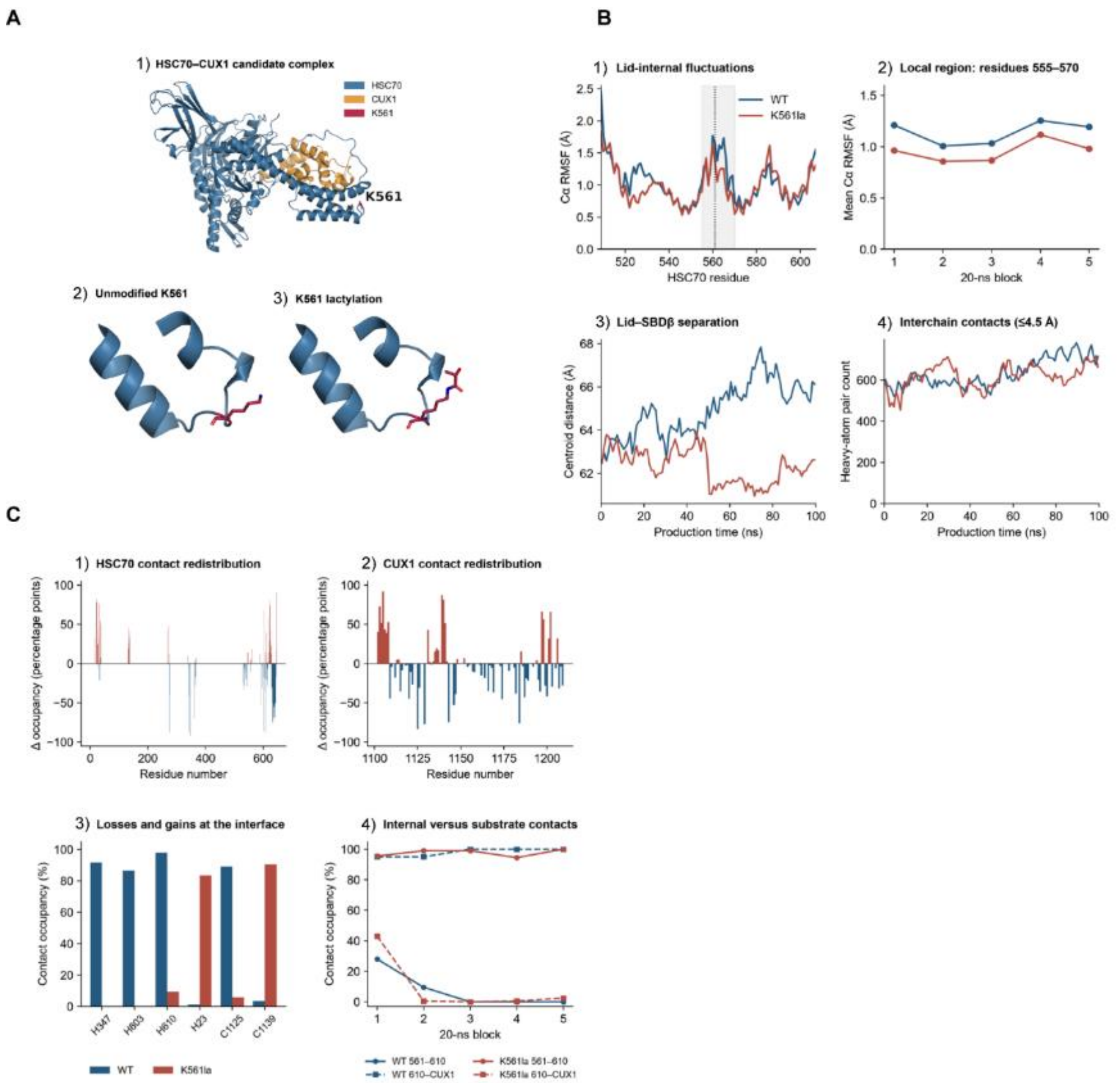

Figure S15 Molecular docking and molecular dynamics simulation results of HSC70 and CUX1

(A) Candidate complex and the K561 lactylation site. 1) WT complex at the simulation preparation stage. HSC70, blue; CUX1 fragment, orange; K561, sticks. 2) 3) Local structures of unmodified and lactylated K561. Carbon, magenta; nitrogen, blue; oxygen, red. The two states were aligned by HSC70 Cα atoms and are shown in the same local orientation. Images were generated from the prepared structures; both states were derived from the same candidate docking pose.

(B) Conformational and interface contact changes upon modification. Blue, WT; red, K561la. 1)

Per-residue Cα RMSF after alignment on the respective lid region; the gray shaded area indicates residues 555-570, and the dashed line marks residue 561. 2) Mean RMSF of residues 555–570 within each of five consecutive 20-ns blocks. 3) Distance between the heavy-atom geometric centers of the lid and SBDβ. 4) Number of intermolecular heavy-atom contacts at a 4.5-Å cutoff. Curves in 3) and 4) are means over continuous 1-ns windows (100 frames per window)

(C) Reduced, increased, and internally rearranged interface contacts. 1) 2) Difference in contact occupancy for all HSC70 and CUX1 residues (K561la - WT); positive and negative values indicate increased and decreased occupancy, respectively. 3) Representative contacts with decreased and increased occupancy. H, HSC70; C, CUX1; numbers indicate residue positions. 4) Block-wise occupancy of the intramolecular 561–610 contact (solid) and the intermolecular 610–CUX1 contact (dashed). Contacts were defined by a heavy-atom distance cutoff of ≤4.5 Å, using 1,000 sampled frames per state across five consecutive 20-ns blocks.
